# A Novel CRISPR Interference Effector Enabling Functional Gene Characterization with Synthetic Guide RNAs

**DOI:** 10.1089/crispr.2022.0056

**Published:** 2022-12-12

**Authors:** Clarence Mills, Andrew Riching, Ashleigh Keller, Jesse Stombaugh, Amanda Haupt, Elena Maksimova, Sarah M. Dickerson, Emily Anderson, Kevin Hemphill, Chris Ebmeier, John A. Schiel, Josien Levenga, Matthew Perkett, Anja van Brabant Smith, Zaklina Strezoska

**Affiliations:** ^1^ Horizon Discovery, a PerkinElmer Company, Lafayette, Colorado, USA and University of Colorado-Boulder, Boulder, Colorado, USA.; ^2^ Mass Spectrometry Core Facility, University of Colorado-Boulder, Boulder, Colorado, USA.

## Abstract

While CRISPR interference (CRISPRi) systems have been widely implemented in pooled lentiviral screening, there has been limited use with synthetic guide RNAs for the complex phenotypic readouts enabled by experiments in arrayed format. Here we describe a novel deactivated Cas9 fusion protein, dCas9-SALL1-SDS3, which produces greater target gene repression than first or second generation CRISPRi systems when used with chemically modified synthetic single guide RNAs (sgRNAs), while exhibiting high target specificity. We show that dCas9-SALL1-SDS3 interacts with key members of the histone deacetylase and Swi-independent three complexes, which are the endogenous functional effectors of SALL1 and SDS3. Synthetic sgRNAs can also be used with *in vitro*-transcribed dCas9-SALL1-SDS3 mRNA for short-term delivery into primary cells, including human induced pluripotent stem cells and primary T cells. Finally, we used dCas9-SALL1-SDS3 for functional gene characterization of DNA damage host factors, orthogonally to small interfering RNA, demonstrating the ability of the system to be used in arrayed-format screening.

## Introduction

Methods for genetic loss-of-function (LOF) experiments are indispensable in cell biology for understanding gene function and are essential to the modern drug discovery pipeline. The discovery of small interfering RNAs (siRNAs) made possible the systematic interrogation of the entire human coding genome through LOF studies shortly after its draft sequence was determined in the early 2000s.^1^ Arrayed LOF screens have identified druggable gene targets and allowed for screens using complex phenotypic readouts and short timepoint experiments that would be otherwise challenging to perform in pooled format experiments.^2–15^ A significant component of functional gene characterization, particularly in a screening workflow context, is downstream validation of relevant gene targets, for which orthogonal modulation techniques are especially valuable.^8,16^

In the last decade, CRISPR-Cas9 has democratized the field of gene editing as an RNA-guided DNA-cleaving enzyme, with the predominant outcome being the disruption of gene function by causing small insertions and deletions (indels) at the targeted DNA cut site.^17–20^ CRISPR-Cas9 has proven to be a valuable LOF technology that complements earlier methods, and it is especially valuable for long-term generation of knockout cells or animal models.^21,22^ Derived CRISPR modulation systems have also been developed which rely on nuclease-deactivated Cas9 (dCas9), most notably CRISPR activation (CRISPRa) and CRISPRi.^23–27^ These techniques use dCas9 to bring either transcriptional activators or transcriptional repressors, respectively, to a transcriptional start site (TSS) and target endogenous genes for expression modulation at the transcriptional level versus gene disruption via formation of indels.

First generation CRISPRi systems relied upon dCas9 fusion to the transcriptional repressor domain Krüppel associated box (KRAB). CRISPRi was found to be highly specific and easily programmable and has been successfully used for functional gene characterization.^25,28,29^ Unlike CRISPR knockout (CRISPRko), CRISPRi is a truly orthogonal approach to siRNA as it downregulates native expression during transcription, whereas siRNA degrades mRNA transcripts.^30^ CRISPRi also offers some additional, distinct ways to target lncRNAs, and to mediate isoform-specific gene knockdown.^31–33^ In addition, unlike CRISPRko, CRISPRi does not result in a permanent, variable, and heterogeneous change in the DNA across a cell population. Although CRISPRi has been successfully leveraged for pooled screening, current CRISPRi systems do not always produce sufficient repression that results in robust phenotypes.^28,34–39^

Second generation systems, such as dCas9- KRAB-Methyl-CpG Binding Protein 2 (MeCP2), have been developed to enhance the levels of transcriptional repression through screening efforts to characterize additional repressor domains.^40–43^ However, reports of CRISPRi with synthetic guide RNAs to mediate transcriptional repression, particularly for arrayed LOF studies, are limited.^44–46^ Enabling broad use of CRISPRi with synthetic guide RNAs would facilitate functional gene characterization in arrayed formats, including the added power of complex phenotypic readouts.

To identify a more potent CRISPRi system amenable to use with synthetic single guide RNAs (sgRNAs), we performed an arrayed screen using a small set of novel repressor domains. We identified domains from Sal-like protein 1 (SALL1) and Sin3a corepressor complex component (SDS3) to be the most effective repressors among those tested, and we used these domains to engineer a bipartite repressor dCas9 fusion. dCas9-SALL1-SDS3 significantly improves CRISPR-mediated repression compared to dCas9-KRAB and dCas9-KRAB- MeCP2, the most widely used first- and second-generation CRISPRi effectors. This novel SALL1-SDS3 repressor exhibits higher levels of target gene repression while retaining high target specificity.

We find that dCas9-SALL1-SDS3 interacts with key members of the histone deacetylase (HDAC) and Swi-independent 3 (Sin3) complexes, which are the native binding partners and endogenous functional effectors of these domains. We demonstrate that *in vitro*-transcribed dCas9-SALL1-SDS3 mRNA can be co-delivered with synthetic sgRNAs, providing a simple workflow to achieve potent gene repression in clinically relevant cell types such human induced pluripotent stem cells (hiPSCs) and primary human T cells. Finally, we show that dCas9-SALL1-SDS3 can be used orthogonally to siRNA for functional gene characterization in arrayed assays.

## Materials and Methods

### dCas9-repressor plasmid construction and generation of stable cell lines

Sequence- and ligation-independent cloning was used to join repressor domains (Supplementary Table S1) to the C-terminus of a human codon-optimized *Streptococcus pyogenes* dCas9 (containing an N-terminal 1 × FLAG tag and two nuclear localization signals) via an 11 amino acid glycine-serine linker, and to insert the construct into a lentiviral expression vector.^47^ Type V dCas-SALL1-SDS3 fusions were cloned into a similar lentiviral system with the same 11 amino acid glycine-serine linker placed between deactivated MAD7 (derived from dErCas12a) or deactivated dCasΦ-8 (dCas12j) and the SALL1-SDS3 construct. Lentiviral vectors were packaged into lentiviral particles using the Trans-Lentiviral Packaging Kit (Horizon, TLP5919).^48,49^

U2OS Ubi[G76V]-enhanced green fluorescence protein (EGFP) (BioImage, discontinued), U2OS (ATCC; HTB-96), A549 (ATCC; CCL-185), A375 (ATCC; CRL-161, K-562 (ATCC; CCL-243), WTC-11 hiPSCs (Coriell Institute; cat no. GM25256), and Jurkat (ATCC; TIB-152) cells were transduced at a multiplicity of infection of 0.3 with lentiviral particles co-expressing the blasticidin resistance gene and various dCas effectors. Cells were subsequently cultured in cell-line-specific medium containing 5–10 μg/mL blasticidin for a minimum of 10 days to select for cells stably expressing CRISPRi proteins.

### Synthesis of guide RNAs

All guide RNAs were synthesized at Horizon, a PerkinElmer company (formerly Dharmacon™) sgRNAs were designed using the 2016 CRISPRi version 2.1 (v2.1) sgRNA prediction algorithm.^50^ Unless otherwise stated, experiments utilized sgRNAs with two 2′-O-methyl phosphorothioate (MS) modifications at the 5′ and the 3′ end of an sgRNA, for increased nuclease stability, delivered as an equimolar pool of the top three algorithmically ranked sgRNAs (Supplementary Table S2).^51^ The same targeting sequences were used for the synthetic sgRNA and expressed sgRNA comparisons with the exception that in the instances where the spacer does not start with a G, the first base in expressed sgRNA is replaced with a G for transcription initiation.^34,50,52^

### Lipid transfections

U2OS, A549, and A375 cells were seeded in 96-well plates at 10,000, 15,000, or 20,000 cells per well, respectively, 1 day before transfection. Cells were transfected with gene-specific synthetic guide RNAs and siRNAs at a final concentration of 25 nM. Purified plasmids expressing a single gene-specific sgRNA under the control of the human U6 promoter were delivered at 100 ng/well. Synthetic sgRNAs or siRNAs were complexed with DharmaFECT 4 Transfection Reagent (Horizon; T-2005-01) for each experiment in serum-free medium for 20 min. In cases where the cells were not stably expressing a dCas9 CRISPRi construct, dCas9-SALL1-SUDS3 mRNA was co-delivered with sgRNA at 0.2 μg/well using DharmaFECT Duo Transfection Reagent (Horizon; T-2010). sgRNA-expressing plasmids were complexed with DharmaFECT kb Transfection Reagent (Horizon; T-2006) in serum-free medium for 10 min. Complete serum media was added to the transfection mixture and used to replace the medium on the plated cells. The cells then were incubated at 37°C with 5% CO_2_ for the length of time listed in each figure.

### Nucleofections

K562, Jurkat, WTC-11 hiPSCs, and primary human CD4^+^ T cells were nucleofected using the 4D-Nucleofector^®^ X Unit (Lonza) under the conditions listed in Supplementary Table S3. In cases where the cells were not stably expressing a dCas9 CRISPRi construct, dCas9-SALL1-SDS3 mRNA (Horizon; CAS12224) was co-delivered with sgRNA at cell-line-dependent concentrations. WTC-11 hiPSCs were cultured in media containing 10 μM ROCK Inhibitor (STEMCELL; 72302) for 2–4 h before nucleofection to promote survival. CD4^+^ T cells were stimulated with CD3/CD28 Dynabeads (ThermoFisher; 11131D) in media containing 200 U/mL IL-2 (BioLegend; 791902) 5 ng/mL IL-7 (Gibco; PHC0071), and 5 ng/mL IL-15 (BioLegend; 570302) for 72 h before nucleofection.

### Proteasome assay

Experiments were performed in U2OS Ubi[G76V]-EGFP cells stably expressing dCas9 effectors. Cell media was replaced with Dulbecco's Phosphate Buffered Saline 72 h post-transfection and EGFP fluorescence was measured using an EnVision^®^ Plate Reader (PerkinElmer). Fluorescent values of cell populations transfected with synthetic sgRNAs targeting critical proteasome genes were normalized to fluorescent values of the untreated cell populations.

### Reverse transcription-quantitative polymerase chain reaction

Total RNA was extracted in a guanidine thiocyanate buffer and isolated with Wizard^®^ SV 96 Binding Plates (Promega; A2271). cDNA was subsequently reverse-transcribed using Maxima First Strand cDNA Synthesis Kit for reverse transcription-quantitative polymerase chain reaction (RT-qPCR), with dsDNase (ThermoFisher; K1672). qPCR was performed using TaqMan Gene Expression Master Mix (Applied Biosystems; 4369016) and TaqMan Gene Expression Assays (Supplementary Table S4) using a LightCycler 480 II (Roche). The relative expression of each gene was calculated with the ΔΔCq method using *GAPDH* or *ACTB* as the housekeeping gene and normalized to a non-targeting control (NTC).

### Cas9 enzyme-linked immunosorbent assay

Duplicate 1,000,000 cell aliquots were lysed in RIPA buffer supplemented with Protease Inhibitor Cocktail (ThermoFisher; 87786). Lysates were diluted 1:3 in assay diluent, loaded on anti-Cas9 antibody-coated plates (Cell Biolabs; PRB-5079) and processed per the manufacturer's protocol.

### Whole-transcriptome RNA sequencing

U2OS cells stably expressing either dCas9-KRAB or dCas9-SALL1-SDS3 were transfected with synthetic sgRNAs, and Wild-type (WT) U2OS cells were transfected with gene target-matched siRNAs to control for pathway-related effects of gene knockdown. Total RNA was isolated 48 h post-transfection as described above. Libraries were prepared from 1.2 μg of purified RNA and sequenced on an Illumina HiSeq platform (Azenta).

Sequences were processed through the Illumina DRAGEN RNA Pipeline v3.7.5 to quantify transcripts per million and read counts. Differential expression analysis was then performed using DESeqv1.38.0.^53^ Off-target alignments were performed against hg38 allowing for up to two flaws (mismatches) in the protospacer adjacent motif-proximal seed region and up to two flaws in the non-seed region for a total of up to four flaws per potential sgRNA binding site. Custom Python scripts were used to identify genomic regions within 500 bp of TSS.

### Co-immunoprecipitation

WT U2OS cells or U2OS cells stably expressing dCas9, dCas9-KRAB, or dCas9-SALL1-SDS3 were plated in 15 cm dishes and transfected with synthetic sgRNAs as described above. Nuclei were extracted using the NE-PER Nuclear and Cytosolic Extraction kit (ThermoFisher; 78835) supplemented with protease inhibitor (ThermoFisher; 1862209) 48 h post-transfection. Protein concentrations of nuclear lysates were determined by BCA assay (ThermoFisher; 23225). Two hundred fifty micrograms aliquots of nuclear lysates were diluted with Co-immunoprecipitation (Co-IP) buffer (150 mM NaCl, 50 mM Tris-Cl pH 7.4, 1 mM ethylenediaminetetraacetic acid, 1% Triton X-100) and 5% of the total lysate volume was removed and reserved for input samples. The remaining lysate was incubated with α-FLAG-conjugated magnetic beads (Sigma; M8823-1ML) overnight at 4°C on an end-over-end mixer.

### Mass spectrometry analysis

Co-IP lysates were prepared as above using 40 μL α-FLAG-conjugated magnetic beads. Following Co-IP, samples were resuspended in Co-IP buffer with reduced Triton X-100 (0.1%). To prepare samples for mass spectrometry, FLAG-tagged affinity purifications were eluted, reduced, and alkylated using 5% (w/v) sodium dodecyl-sulfate, 10 mM tris(2-carboxyethylphosphine), 40 mM 2-chloroacetamide, 50 mM Tris-HCl, pH 8.5, boiled for 10 min, and incubated shaking at 1000 rpm at 37°C for 30 min. Affinity-purified proteins were digested using the SP3 method with Sera-Mag™ carboxylate-functionalized SpeedBeads (Cytiva).^54^ Cleaned-up peptides were then dried in a SpeedVac vacuum concentrator and stored at −20°C until analysis.

Tryptic peptides were suspended in 3% (v/v) acetonitrile, 0.1% (v/v) trifluoroacetic acid and directly injected onto a reversed-phase C18 1.7 μm, 130 Å, 75 mm × 250 mm M-class column (Waters), using an Ultimate 3000 nanoUPLC (ThermoFisher). Peptides were eluted with an acetonitrile gradient and detected using a Q-Exactive HF-X mass spectrometer (ThermoFisher). Raw files were searched against the Uniprot Human database UP000005640 using MaxQuant v.1.6.14.0. All peptide and protein identifications were thresholded at a 1% false discovery rate. Statistical analysis was performed on log2-transformed iBAQ intensities using limma.

### Western blot

Co-IP lysates were prepared as above, using 10 μL α-FLAG-conjugated magnetic beads. Following Co-IP, magnetic beads were resuspended in 20 μL buffer containing 1 × NuPAGE LDS sample buffer (ThermoFisher; NP0007) and 1 × NuPAGE sample reducing agent (ThermoFisher; NP0009) and boiled for 15 min. Proteins were resolved with electrophoresis and transferred to nitrocellulose membranes. Membranes were blocked for 30 min and incubated with primary antibodies against HDAC1 (Cell Signaling; 34589), HDAC2 (Cell Signaling; 2540), RBBP4 (Abcam; ab1765), or HRP-conjugated FLAG (Sigma; A8592-.2MG). Where applicable, membranes were then incubated with goat α-rabbit HRP-conjugated secondary (ThermoFisher; A16096) or goat α-rabbit IR800 secondary (Li-Cor; 926-32211). Chemiluminescence signal was developed with Western Lightning ONE Pico or Femto chemiluminescent substrates (PerkinElmer; NEL131001EA or NEL141001EA). Chemiluminescent and fluorescent signal was imaged with the iBright 1500FL imaging system (ThermoFisher).

### Flow cytometry analysis

At 24 and 72 h post-nucleofection, cells were resuspended in a 1:50 solution of Fc block (BD Biosciences; cat no. 564220) and co-stained with Alexa Fluor 488-conjugated CD4 (BioLegend; 50166932) and APC-conjugated CXCR3 (BioLegend; 353707) antibodies. Unstained, untreated cells were used to gate for CD4^+^ and CXCR3-positive (CXCR3^+^) cells. The percentage of CXCR3^+^ positive cells in the targeted populations was normalized to that in the control populations to determine protein knockdown.

### DNA damage response assays

A549 cells stably expressing dCas9-SALL1-SDS3 were plated at 4000 cells per well and transfected as described above. For each gene target, equimolar pools of siRNA or equimolar pools of synthetic CRISPRi sgRNAs were delivered at a final concentration of 25 nM. At 72 h post-transfection, cells were fixed, permeabilized, blocked, and stained with a primary antibody targeting phospho-histone H2A.X (Ser139) (Invitrogen; MA1-2022) and an Alexa Fluor 488 conjugated fluorescent secondary antibody (Invitrogen; A-11001). Hoechst stain was used to identify nuclei. Cells were imaged with a Celigo Image Cytometer (Nexcelom) and the instrument's masking and cell analysis software was used identify and quantify the nuclei that were DAPI and FITC positive in each well. Cells containing nuclei that were both DAPI and FITC positive were considered to be phospho-H2AX positive and represented as a percentage of all DAPI positive cells in the well.

### Statistical analysis

GraphPad Prism 9 (Dotmatics) was used for statistical analysis. The use of parametric tests was determined with the Shapiro-Wilk test for normality and one- or two-way analysis of variance (ANOVA) were applied. Significant effects were followed by Turkey's or Šídák's *post hoc* tests for multiple comparisons, as detailed in the respective figure legends. Two-way repeated measures ANOVA was applied to time course datasets with the Geisser-Greenhouse correction used to adjust for lack of sphericity. All statistical tests were two-tailed with *p* < 0.05 considered as statistically significant.

## Results

### Identification of a novel potent effector for CRISPRi applications

Our initial testing of the dCas9-KRAB system with synthetic guide RNAs resulted in moderate, transient repression that did not produce a marked phenotype compared to delivery of siRNA reagents (Supplementary Fig. S1A), suggesting a more potent repressor may be needed for a functional CRISPRi system with synthetic guide RNAs. Recent publications have demonstrated that CRISPRi activity can be improved through tethering more active repressor domains to dCas9 and we reasoned that a similar strategy could be implemented to develop a dCas9-based repressor system that can support efficient and transient transcriptional repression using synthetic guide RNAs.^40–43^

**FIG. 1.**
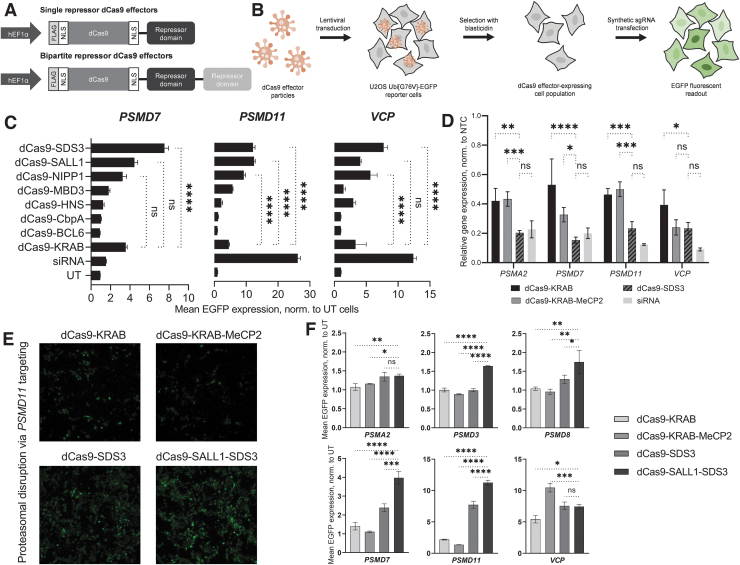
Identification of a potent effector for CRISPRi applications. **(A)** Schematic of single and bipartite repressor dCas9 effectors. **(B)** Schematic of dCas9 effector screening process. Lentiviral particles were used to generate populations of U2OS Ubi[G76V]-EGFP cells that stably expressed the dCas9 effectors. Cells were subsequently transfected with synthetic sgRNAs targeting proteasomal genes and analyzed for EGFP fluorescence. Disruption of proteasome pathway genes inhibits degradation of Ubi-EGFP resulting in an accumulation of EGFP-fluorescence. **(C)** Mean EGFP fluorescence induced by targeting candidate proteasome-related genes *PSMD7*, *PSMD11*, and *VCP* with synthetic sgRNAs in U2OS Ubi[G76V]-EGFP cells stably expressing dCas9 fused to single repressor domains. siRNA delivery was used as a positive control for fluorescence induction. Mean fluorescence was measured 72 h post-transfection and is shown relative to UT cells. **(D)** Relative mRNA expression of proteasome-related genes *PSMA2, PSMD7, PSMD11,* and *VCP* 72 h post-sgRNA or siRNA transfection in U2OS cells stably expressing dCas9-KRAB, dCas9-KRAB-MeCP2, or dCas9-SDS3. All data were normalized to the corresponding NTCs. **(E)** Representative imaging of U2OS Ubi[G76V]-EGFP cells stably expressing dCas9-KRAB, dCas9-KRAB-MeCP2, dCas9-SDS3, or dCas9-SALL1-SDS3 72 h post-transfection of synthetic sgRNAs targeting *PSMD11*. **(F)** Mean EGFP fluorescence induced by targeting proteasomal genes *PSMA2, PSMD3*, *PSMD8, PSMD7, PSMD11,* and *VCP* with synthetic sgRNAs in Ubi[G76V]-EGFP cells stably expressing dCas9-KRAB, dCas9-KRAB-MeCP2, dCas9-SDS3, or dCas9-SALL1-SDS3. Mean fluorescence was measured 72 h post-transfection and is shown relative to UT cells. *n* = 3 biological independent replicates per group. All data presented as mean ± SD. *, **, ***, and ****p < 0.05, 0.01, 0.001, and 0.0001, respectively by two-way ANOVA followed by Tukey's *post hoc* test for multiple comparisons. ANOVA, analysis of variance; dCas9, deactivated Cas9; EGFP, enhanced green fluorescence protein; KRAB, Krüppel associated box; MeCP2, methyl-CpG binding protein 2; NTCs, non-targeting controls; SALL1, Sal-like protein 1; SD, standard deviation; SDS3, Sin3a corepressor complex component; sgRNAs, single guide RNAs; siRNA, small interfering RNA; UT, untransfected.

We hypothesized that transcriptional co-repressors which interact closely with DNA binding proteins in their native context would be well suited for CRISPRi as they could recruit DNA-binding transcription factors and epigenetic modulators but would be less likely to direct the dCas9 fusion to genomic loci in a guide RNA-independent manner. We identified a set of seven domains from various eukaryotic and prokaryotic proteins, all of which were <1 kb in size, to enable efficient lentiviral packaging (Supplementary Table S1). Each domain, along with the KRAB domain from ZNF10, was individually fused to the C-terminus of dCas9 through a flexible linker to create single repressor dCas9 effectors (Fig. 1A).

To evaluate dCas9 effector activity with these domains, we used a reporter cell line previously used to characterize other LOF technologies.^55^ These U2OS cells stably express EGFP fused to a non-cleavable mutant ubiquitin moiety; disruption of the proteasome complex or pathway inhibits degradation of Ubi-EGFP and results in an accumulation of fluorescence.^56^ This proteasome reporter assay enabled us to assess repressor activity against multiple endogenous, distally located genes in a high-throughput manner. To minimize transfection-dependent and clonal effects, we used lentiviral integration to generate stochastic populations of cells that stably expressed each repressor construct.

We interrogated each cell line with chemically-stabilized synthetic sgRNAs designed for CRISPRi using a published sgRNA prediction algorithm (Fig. 1B).^50^ We targeted four genes critical to proteasome function: *PSMA2*, *PSMD7*, *PSMD11*, and *VCP*. Three of the repressor domains, SALL1, SDS3 and NIPP1, each caused a more pronounced phenotype (increase of EGFP fluorescence signal) than dCas9-KRAB for three out of the four target genes tested (Fig. 1C). Knockdown of *PSMA2* did not produce a pronounced phenotypic response by any of the repressors or siRNA (Supplementary Fig. S1B). One effector, dCas9-SDS3, led to significantly greater proteasomal disruption than dCas9-KRAB when targeting *PSMD7* (2.1-fold), *PSMD11* (2.6-fold), or *VCP* (2.3-fold). In comparison, control transfection of siRNAs targeting *PSMD11* and *VCP* resulted in 5.7-fold and 3.7-fold greater mean EGFP expression than that observed with dCas9-KRAB, while siRNAs targeting *PSMD7* produced 2.3-fold less EGFP expression than dCas9-KRAB repression of *PSMD7*.

We next compared the transcriptional repression induced by dCas9-SDS3 to dCas9-KRAB, dCas9-KRAB-MeCP2, and siRNA knockdown, by RT-qPCR analysis of the proteasome gene targets. dCas9-SDS3 significantly enhanced repression of all four tested genes compared to dCas9-KRAB and three out of the four genes compared to dCas9-KRAB-MeCP2 (Fig. 1D) and was used as a benchmark in the next phase of identifying bipartite dCas9-repressors fusions. siRNAs still mediated the most potent knockdown of *PSMD11* and *VCP,* which was consistent with the previously observed phenotypes (Fig. 1C), and comparable knockdown of *PSMA2* and *PSMD7* to dCas9-SDS3.

To further enhance CRISPR-mediated repression, we used the three most active repressor domains (SDS3, SALL1, and NIPP1) in different combinations to create 10 bipartite repressor dCas9 effectors and evaluated them in the same reporter cell line. While most of the bipartite fusions produced less pronounced repressive effects compared to dCas9-SDS3, one bipartite repressor combination, dCas9-SALL1-SDS3, produced a more robust phenotype in two of the three targeted genes (Supplementary Fig. S1C). In light of previous reports that repressor domain position can impact CRISPRi effects, we next tested whether repressor placement at the N-terminus of Cas9 could further improve CRISPR-mediated repression.^42^ We found that the most robust CRISPRi effects were produced with the C-terminal fusion of SALL1-SDS3 to dCas9, and that fusion of the KRAB domain to the N-terminus of dCas9 did not produce discernible improvements in CRISPRi-mediated proteasomal disruption (Supplementary Fig. S1D).

Finally, we compared the repressive effects mediated by dCas9-SALL1-SDS3 to that mediated by dCas9-KRAB, dCas9-SDS3, and dCas9-KRAB-MeCP2 using synthetic sgRNAs targeting six proteasomal genes (Fig. 1E, F) and observed significantly enhanced phenotypes with dCas9-SALL1-SDS3 in four out of the six targeted genes compared to dCas9-SDS3, five out of the six targeted genes compared to dCas9-KRAB-MeCP2, and in all six of the targeted genes compared to dCas9-KRAB.

### Robust, specific repression with dCas9-SALL1-SDS3 and synthetic sgRNAs

**FIG. 2.**
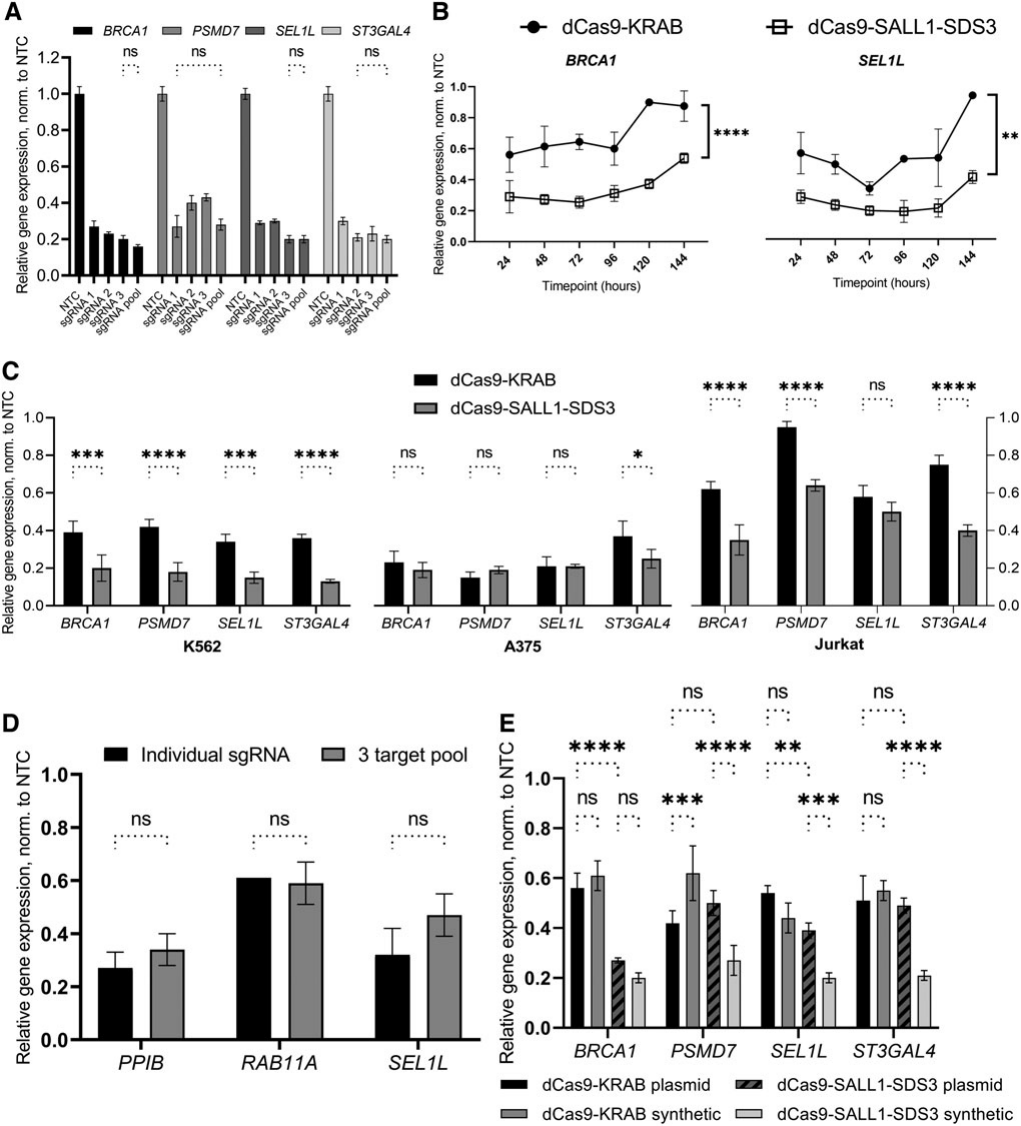
Robust transcriptional repression with dCas9-SALL1-SDS3 and synthetic guide RNAs. **(A)** Relative mRNA expression of *BRCA1, PSMD7, SEL1L,* and *ST3GAL4* 72 h post-sgRNA transfection in U2OS cells stably expressing dCas9-SALL1-SDS3. Cells were either transfected with individual sgRNAs targeting the respective gene or an equimolar pool of three individual sgRNAs. All data were normalized to the corresponding NTC sgRNAs. **(B)** Comparison of relative mRNA expression of *BRCA1* and *SEL1L* in U2OS cells stably expressing either dCas9-SALL1-SDS3 or dCas9-KRAB 24, 48, 72, 96, 120, and 144 h post-transfection of synthetic sgRNAs targeting the respective genes. All data were normalized to the corresponding NTCs. Repeated measures ANOVA was performed with ** and ****p < 0.01 and 0.0001, respectively. **(C)** Relative mRNA expression of *BRCA1, PSMD7, SEL1L,* and *ST3GAL4* 72 h post-synthetic sgRNA delivery in K562, A375, and Jurkat cells expressing either dCas9-KRAB or dCas9-SALL1-SDS3. All data were normalized to the corresponding NTCs. Two-way ANOVA was followed by Šídák's *post hoc* test for multiple comparisons. **(D)** Comparison of relative mRNA expression of *PPIB, RAB11A,* and *SEL1L* in hiPSCs 72 h post-transfection of either individual synthetic sgRNAs targeting the listed gene or a multiplexed pool of sgRNAs targeting all three genes simultaneously. All data were normalized to the corresponding NTCs. **(E)** Comparison of relative mRNA expression of *BRCA1, PSMD7, SEL1L,* and *ST3GAL4* in U2OS cells stably expressing dCas9-KRAB or dCas9-SALL1-SDS3 72 h post-transfection of either individual synthetic sgRNAs or matched sgRNA-expressing plasmids. All data were normalized to the corresponding NTCs. *n* = 3 biologically independent replicates per group. All data presented as mean ± SD. *, **, ***, and ****p < 0.05, 0.01, 0.001, and 0.0001, respectively by two-way ANOVA followed by Tukey's *post hoc* test for multiple comparisons unless otherwise noted. hiPSCs, human induced pluripotent stem cells.

Our previous work with CRISPRa demonstrated that multiple synthetic guide RNAs targeting the same TSS can be combined and delivered as a pool to effect greater transcriptional activation.^52^ To test whether the pooling of synthetic sgRNAs can also enhance CRISPR-mediated repression, we compared the repressive activity of individual sgRNAs to an equimolar pool of three. While the pooling of sgRNAs did not significantly increase target gene repression, the pools repressed each gene to approximately the level observed with the most active individual guide in the pool (73–80% repression) (Fig. 2A).

We targeted 25 genes in U2OS cells stably expressing dCas9-SALL1-SDS3 with pooled synthetic sgRNAs and observed consistent levels of transcriptional repression that did not correlate to the level of basal gene expression (Supplementary Fig. S2A, B), as has been reported with CRISPRa system.^52^ Thus, we opted to proceed with pooling as a strategy to attenuate any variability observed in individual sgRNA activity. The ability to pool sgRNA designs provides an additional benefit of reducing experimental size and complexity.

We then performed a time course to examine the duration of CRISPR-mediated repression with synthetic sgRNAs. U2OS cells stably expressing either dCas9-KRAB or dCas9-SALL1-SDS3 were transfected with pools of three synthetic sgRNAs targeting genes outside the proteasome pathway and transcriptional repression was assessed every 24 h for 6 days (Fig. 2B, Supplementary Fig. S2C). dCas9-SALL1-SDS3 mediated significantly greater target gene repression than dCas9-KRAB throughout the duration of the study with the differences being particularly pronounced at early and late timepoints. Notably, for each gene targeted, the maximal observed level of repression was substantially higher in cells expressing dCas9-SALL1-SDS3 (78–81%) than those expressing dCas9-KRAB (44–67%).

Immunofluorescent analysis indicated that the increased transcriptional repression observed with dCas9-SALL1-SDS3 resulted in a higher degree of protein knockdown compared to dCas9-KRAB (Supplementary Fig. S2D, E). We observed similar levels of dCas9-effector expression in both cell lines, confirming that the enhanced transcriptional repression observed with dCas9-SALL1-SDS3 was not resulting from increased effector expression (Supplementary Fig. S2F).

To ensure that this novel CRISPRi repressor was active in cell lines other than U2OS, we generated several cell lines (A375, Jurkat, and K562) that stably expressed dCas9-SALL1-SDS3 and interrogated them with pooled synthetic sgRNAs targeting proteasome and non-proteasome gene targets. We observed significantly enhanced target gene repression in K562 and Jurkat cells expressing dCas9-SALL1-SDS3 than in those expressing dCas9-KRAB and similarly robust levels of target repression in both A375 cell lines (75–81% with dCas9-SALL1-SDS3, 63–85% with dCas9-KRAB) (Fig. 2C), indicating the system is broadly applicable across different cancer cell lines.

We then generated hiPSCs that stably expressed dCas9-SALL1-SDS3 to test whether this approach would be effective in a clinically relevant cell model, and if synthetic sgRNAs targeting different genes could be multiplexed to simultaneously repress three targets (Fig. 2D). For each gene target, the observed transcriptional repression was comparable in cells nucleofected with the multiplexed pool as in those nucleofected with individual sgRNAs demonstrating that synthetic sgRNAs can be combined to simultaneously perturb multiple genes in hiPSCs without the use of an integrated multiplexed vector.^57^

To compare the CRISPRi effects of synthetic gRNAs to those of expressed sgRNAs, we examined the transcriptional repression of target genes in U2OS cells stably expressing dCas9-KRAB or dCas9-SALL1-SDS3 and transfected with either synthetic sgRNAs or sgRNA-expressing plasmids (Fig. 2E). In cells expressing dCas9-SALL1-SDS3, we observed similar or enhanced target gene repression in the populations transfected 72 h prior with synthetic sgRNAs compared to those transfected with plasmid-expressed sgRNAs. Interestingly, the repression with expressed sgRNAs was similar between dCas9-KRAB and the dCas9-SALL1-SDS3 repressor systems, suggesting that the novel system is specifically engineered for improved target gene repression with synthetic sgRNA. In line with this, we see greater target repression with synthetic sgRNAs with the dCas9-SALL1-SDS3 repressor system, while there is comparable, if not lesser, target gene repression with synthetic than plasmid sgRNA transfection in the dCas9-KRAB expressing cells.

**FIG. 3.**
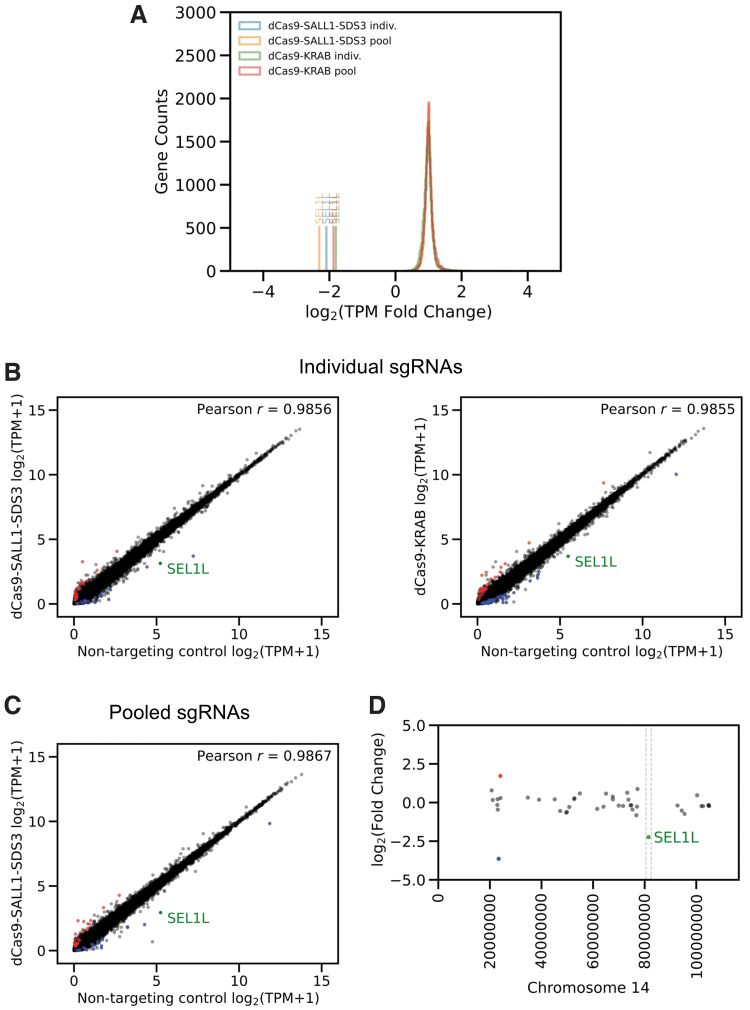
dCas9-SALL1-SDS3 repression is highly target-specific. **(A)** Histogram showing the distribution of global transcriptional expression, represented as TPM, in U2OS cells stably expressing either dCas9-SALL1-SDS3 or dCas9-KRAB that were transfected with either individual (indiv.) or pooled synthetic sgRNAs targeting *SEL1L*. Fold change in *SEL1L* and global transcriptional expression compared to the corresponding NTCNTC is denoted on the x-axis and represented as log_2_ transformed TPM fold change. *n* = 2 biologically independent samples per group. **(B)** Scatter plots of gene expression levels (log_2_ transformed TPM +1, TPM with a pseuudocount of one added before log transformation) in U2OS cells stably expressing dCas9-SALL1-SDS3 (left) or dCas9-KRAB (right) transfected with individual synthetic sgRNA targeting *SEL1L* compared to cells transfected with NTCs (x-axis). DESeq analysis was used to identify up- and down-regulated genes. Up- or down-regulated genes (*p* < 0.05) with absolute log_2_-fold change >1.5 in gene expression (represented as log_2_ transformed TPM +1) marked red and blue, respectively. *r* Indicates Pearson's correlation coefficient, calculated for log-transformed values on all genes except *SEL1L*. **(C)** Scatter plots of gene expression levels in U2OS cells stably expressing dCas9-SALL1-SDS3 transfected with pooled synthetic sgRNAs targeting *SEL1L* compared to cells transfected with NTCs. **(D)** Genomic mapping of significantly differentially expressed genes (*p* < 0.05) across chromosome 14 in U2OS cells stably expressing dCas9-SALL1-SDS3 that were transfected with individual synthetic sgRNA targeting *SEL1L*. Up- or down-regulated genes with absolute log_2_-fold change >1.5 in gene expression (represented as log_2_ transformed TPM +1) marked red and blue, respectively. The 1 Mb region up and downstream of the sgRNA target site is denoted with dotted lines. TPM, transcripts per million.

To evaluate the specificity of dCas9-SALL1-SDS3-mediated transcriptional repression, we performed whole transcriptome RNA sequencing on populations of U2OS cells stably expressing dCas9-SALL1-SDS3 or dCas9-KRAB 48 h post-transfection of individual or pooled synthetic sgRNAs. There were small numbers of significantly differentially expressed genes (absolute log2-fold change >1.5, adjusted *p*-value <0.05) in untransfected (UT) cell expressing either dCas9-KRAB (24) or dCas9-SALL1-SDS3 (33) when compared to the WT cell line (Supplementary Fig. S3A, Supplementary File S1), indicating that dCas9-SALL1-SDS3 expression does not cause widespread non-specific repression. In addition, dCas9-SALL1-SDS3 effected greater repression than dCas9-KRAB against all three gene targets (Fig. 3A, Supplementary Fig. S3B).

DESeq comparison of cells transfected with individual sgRNA targeting *CD46*, *PPIB*, and *SEL1L* respectively to those transfected with a NTC revealed minimal changes in differential expression, indicating highly specific promoter targeting, similar to that of dCas9-KRAB (Fig. 3B, Supplementary Fig. S3C). Furthermore, using pools of three sgRNAs does not have negative effects on CRISPRi specificity as evidenced by the similar Pearson's *r* values (Fig. 3C, Supplementary Fig. S3D) and lower numbers of significantly differentially expressed genes (Table 1).

**Table 1.** Specificity analysis of dCas9-SALL1-SDS3 and dCas9-KRAB targeting

***Gene target***	***Comparison***	***Total significant differentially expressed genes***	***Total number of genes proximal to potential off-target binding sites***	***Significantly differentially expressed genes proximal to potential off-target binding sites***
CD46	dCas9-SALL1-SDS3 indiv sgRNA vs. NTC	53	35	
CD46	dCas9-KRAB indiv sgRNA vs. NTC	66	35	
CD46	dCas9-SALL1-SDS3 pool vs. NTC	36	111	
CD46	siRNA pool vs. siRNA NTC	174	*Orthogonal control*	
PPIB	dCas9-SALL1-SDS3 indiv sgRNA vs. NTC	65	198	**MEST**
PPIB	dCas9-KRAB indiv gRNA vs. NTC	38	198	**MEST;** SCHIP1
PPIB	dCas9-SALL1-SDS3 pool vs. NTC	41	686	**MEST**
PPIB	siRNA pool vs. siRNA NTC	35	*Orthogonal control*	**MEST**
SEL1L	dCas9-SALL1-SDS3 indiv sgRNA vs. NTC	63	295	**LOC102724219;** SULT1A3
SEL1L	dCas9-KRAB indiv sgRNA vs. NTC	94	295	**LOC102724219; SLX1B-SULT1A4**
SEL1L	dCas9-SALL1-SDS3 pool vs. NTC	60	414	**LOC102724951; LOC102724843; LOC102724219**
SEL1L	siRNA pool vs. siRNA NTC	119	*Orthogonal control*	**LOC102724951; LOC102724843; SLX1B-SULT1A4; LOC102724219**

Global transcriptional expression in U2OS cells transfected with target-specific guides was compared to expression in matched cells transfected with NTCs. Up- or down-regulated genes (*p* < 0.05) with absolute log2-fold change >1.5 in gene expression were considered significantly differentially expressed. Off-target alignments were performed against hg38 for each sgRNA sequence allowing for up to two flaws (mismatches) in the PAM-proximal seed region and up to two flaws in the non-seed region for a total of up to four flaws per potential sgRNA binding site; potential off-target sgRNA binding sites were considered proximal to a gene if they were within 500 base pairs of a TSS. The lists of genes proximal to potential off-target binding sites were cross-referenced against the significantly differentially expressed genes in each comparison; those significantly differentially expressed genes proximal to a potential off-target binding site are shown in the right-most column. U2OS cell transfected with target-matched siRNA pools were used to control for pathway-related effects of target gene knockdown. For each gene target, the significantly differentially expressed genes identified in the siRNA comparison were cross-referenced against the significantly differentially expressed genes found to be proximal to a potential off-target sgRNA-binding site; those genes found in both categories are shown in bold. Given the orthogonal nature of siRNA targeting, it is likely that the observed differential expression of these bolded genes arose from target knockdown rather than off-target sgRNA binding.

dCas9, deactivated Cas9; KRAB, Krüppel associated box; NTCs, non-targeting controls; PAM, protospacer adjacent motif; SALL1, Sal-like protein 1; SDS3, Sin3a corepressor complex component; sgRNAs, single guide RNAs; siRNA, small interfering RNA; TSS, transcriptional start site.

The genomic sequences within 500 bp of the TSS of each differentially expressed gene were examined for near-sequence matches to the transfected sgRNAs with a target-matched, siRNA-transfected sample used to control for potential pathway-related effects of target gene knockdown (Table 1). Only one differentially expressed gene in transfected dCas9-SALL1-SDS3 expressing cells, *SULT1A3*, could be explained by potential non-specific binding of the sgRNA suggesting that the vast majority of the observed differentially expressed genes did not result from non-specific dCas9-SALL1-SDS3 targeting. Importantly, no genes within 1Mb of the target loci were significantly up or down-regulated (Fig. 3D, Supplementary Fig. S3E) indicating that the repressive effects of dCas9-SALL1-SDS3 binding do not spread to neighboring genes across the topologically associating domain, or physically self-interacting DNA region.

To explore if this new bipartite repressor could be used with other CRISPR Cas enzymes, we fused SALL1-SDS3 to two different type V Cas enzymes, deactivated MAD7 derived from ErCas12a (dMAD7) and deactivated CasΦ/Cas12j (dCasj), and generated U2OS cell lines that stably expressed the respective fusions. These cells, along with U2OS cells stably expressing dMAD7 or dCasΦ alone, were transfected with BRCA1-targeting synthetic guide RNAs designed for each respective Cas protein. At 48 h post-transfection we observed greater transcriptional repression in cells expressing the fusion proteins compared to those expressing the respective deactivated type V Cas protein alone (Supplementary Fig. S4A, B), demonstrating that SALL1-SDS3 can be fused to either Type II or Type V Cas enzymes and used with synthetic guide RNA to achieve targeted transcriptional repression.

### dCas9-SALL1-SDS3 recruits Sin3 and HDAC complex proteins

Given the robust and comparatively longer duration of transcriptional repression observed with dCas9-SALL1-SDS3, we hypothesized that repression was dependent on the recruitment of additional endogenous repressor complexes. dCas9-KRAB is thought to enact transcriptional repression in part through the recruitment of KAP1, resulting in trimethylation on histone 3 lysine 9 (H3K9) proximal to the targeted genomic site.^29,58^ The N-terminal domain of SALL1 has been reported to interact with the nucleosome remodeling and deacetylase, while SDS3 is part of the Sin3 complex.^59–62^ To date neither has been characterized as part of a Cas fusion protein.

**FIG. 4.**
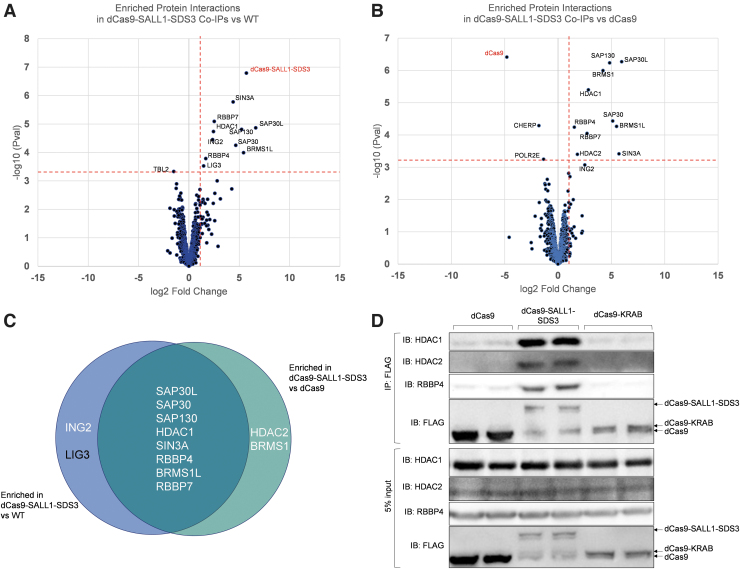
dCas9-SALL1-SDS3 recruits the SIN3A complex. **(A, B)** Volcano plots of enriched protein interactions in dCas9-SALL1-SDS3 versus WT Co-IPs **(A)** or versus dCas9 Co-IPs **(B)**, identified by mass spectrometry. Hits were defined as interactions enriched >2.5-fold (log_2_-fold-change >1.32) in dCas9-SALL1-SDS3 Co-IPs at a significance threshold of *p* < 0.0008 (adjusted *p*-value <0.05), denoted by vertical and horizontal dashed lines, respectively. *n* = 4 (dCas9-SALL1-SDS3 and dCas9) or *n* = 3 (WT) biological replicates per group. dCas9 and dCas9-SALL1-SDS3 are denoted in red. **(C)** Overlap of significantly enriched protein interactions between dCas9-SALL1-SDS3 versus WT and dCas9-SALL1-SDS3 versus dCas9 Co-IP comparisons. White text denotes components of the Sin3 complex. **(D)** Co-IP/Western blot analysis using nuclear extracts from U2OS cells stably expressing FLAG-tagged dCas9, dCas9-SALL1-SDS3, or dCas9-KRAB. Following Co-IP with α-FLAG, proteins were resolved by SDS-PAGE and immunoblotted for HDAC1, HDAC2, RBBP4, and FLAG. *n* = 5 biological replicates per group. Co-IP, Co-immunoprecipitation; SDS-PAGE, sodium dodecyl-sulfate polyacrylamide gel electrophoresis; WT, wild-type.

To investigate the mechanism of repression, we performed an unbiased protein interaction screen. Protein interaction partners were co-precipitated with dCas9-SALL1-SDS3 from U2OS lysates and analyzed by mass spectrometry (Co-IP/MS). Our screen identified eight proteins that were significantly enriched (log 2 FC >1, *p*-value <0.01) in dCas9-SALL1-SDS3 precipitates compared to WT U2OS cells (Fig. 4A, Supplementary Fig. S5A) and U2OS cells stably expressing dCas9 (Fig. 4B, Supplementary Fig. S5B). All eight hits are known members of the Sin3 and HDAC complexes (Fig. 4C). Interestingly, we observed no significant protein interactions with dCas9 and cellular proteins in the lysate (Supplementary Fig. S6A–C), indicating that the prokaryotic protein dCas9 has little interaction with the mammalian proteome.

To confirm the results of the CoIP/MS screen, we immunoblotted the co-precipitations for candidate hits and included co-precipitations from U2OS cells stably expressing dCas9-KRAB. RBBP4, HDAC1, and HDAC2 were clearly detected in co-precipitates from cells expressing dCas9-SALL1-SDS3 but not in those from cells expressing dCas9 (Fig. 4D). This data suggests that dCas9-SALL1-SDS3 mediates transcriptional repression through the recruitment of the Sin3 and HDAC complexes to target loci. Interestingly, RBBP4, HDAC1, and HDAC2 were also not detected in precipitates from cells expressing dCas9-KRAB, although KRAB has been reported to interact with HDAC complexes via its interactions with KAP1.^63–65^

### Potent repression with *in vitro*-transcribed dCas9-SALL1-SDS3 mRNA

**FIG. 5.**
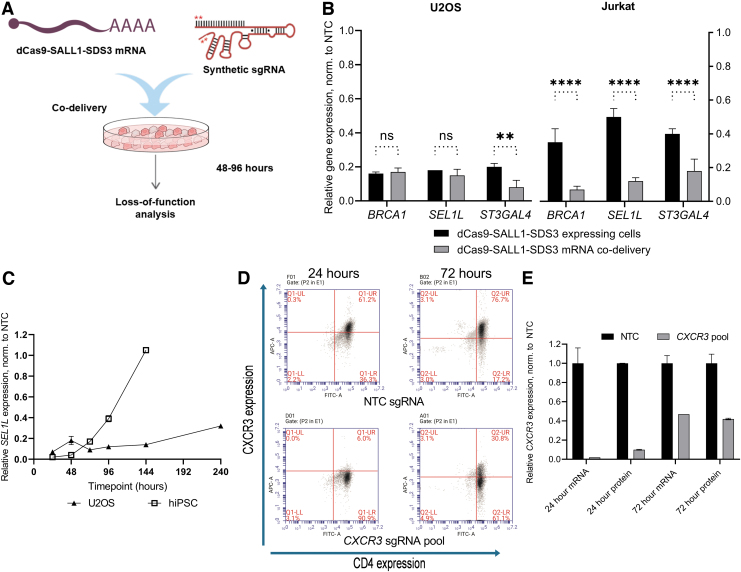
Potent repression with *in vitro*-transcribed dCas9-SALL1-SDS3 mRNA. **(A)** Workflow depicting co-transfection of dCas9-SALL1-SDS3 mRNA and synthetic sgRNA (stars denote 2′-O-methyl phosphorothioate chemical modifications). **(B)** Comparison of relative mRNA expression of *BRCA1*, *SEL1L*, and *ST3GAL4* in U2OS (left) and Jurkat (right) cells stably expressing dCas9-SALL1-SDS3 72 h post-delivery of synthetic sgRNAs, to relative expression in WT U2OS and Jurkat cells in which dCas9-SALL1-SDS3 mRNA was co-delivered with matched synthetic sgRNAs. All data were normalized to the corresponding NTC sgRNAs. **(C)** Relative mRNA expression of *SEL1L* 24, 48, 72, 96, 144, and 240 h post-delivery of dCas9-SALL1-SDS3 mRNA and synthetic sgRNAs into U2OS or hiPSCs. All data were normalized to the corresponding NTCs. **(D)** Representative flow cytometry plots showing *CXCR3* expression in primary human CD4^+^ T-cells 24 h (left) or 72 h (right) after co-nucleofection of dCas9-SALL1-SDS3 mRNA and pooled synthetic sgRNAs targeting *CXCR3* (bottom) or NTCs (top). Untreated, unstained cells were used for gating. *n* = 2 biologically independent samples. **(E)** Relative mRNA and protein expression of *CXCR3* 24 and 72 h post-nucleofection of primary human T cells with dCas9-SALL1-SDS3 mRNA and synthetic sgRNAs. All data were normalized to the corresponding NTCs. *n* = 2 biologically independent samples. *n* = 3 biologically independent replicates per group unless otherwise stated. All data presented as mean ± SD. ** and ****p < 0.01 and 0.0001, respectively by two-way ANOVA followed by Tukey's *post hoc* test for multiple comparisons.

Generating a cell line that stably expresses a dCas9 effector can be time-consuming and is not always feasible with some therapeutically relevant models such as primary immune cells.^66,67^ To enable non-integrative delivery of the dCas9 effector alongside synthetic sgRNAs, we generated *in vitro*-transcribed dCas9-SALL1-SDS3 mRNA and co-transfected the mRNA and synthetic sgRNA into cells to assess gene repression (Fig. 5A).

We compared mRNA and synthetic sgRNA co-delivery to delivery of synthetic sgRNAs into cells stably expressing dCas9-SALL1-SDS3 in both U2OS and Jurkat cells (Fig. 5B). Target gene repression in co-transfected WT U2OS cells was comparable to that observed in the transfected stable cell populations 72 h post-transfection. However, co-nucleofection of dCas9-SALL1-SDS3 mRNA alongside sgRNA in WT Jurkat cells resulted in significantly greater target repression than delivery of sgRNA in stable cell populations (>80% repression vs. 50–65% repression). This surprising difference in efficacy could be due to the relatively low level of dCas9-SALL1-SDS3 expression in the Jurkat stable line (Supplementary Fig. S7A) and highlights the challenges of generating effective stable cells in some cell types.

To characterize the temporal effects of dCas9-SALL1-SDS3 mRNA-mediated repression, we performed a time course in both U2OS cells and hiPSCs. Target gene repression was most robust 24–48 h after delivery in both cell types (Fig. 5C). In U2OS cells, >60% target repression persisted for at least 144 h post-transfection (Supplementary Fig. S7B) whereas in hiPSCs, target gene repression had fully recovered to basal levels by that timepoint (Supplementary Fig. S7C). This difference could be due to the highly proliferative nature of hiPSCs; with frequent cell doublings, the concentrations of sgRNA and dCas9-SALL1-SDS3 mRNA likely decrease below functional levels more rapidly. Importantly, co-nucleofection of dCas9-SALL1-SDS3 mRNA and synthetic sgRNAs into hiPSCs did not appear to impact pluripotency (Supplementary Fig. S7D, E).

To test whether this approach could be applied to primary immune cells, we nucleofected primary human CD4^+^ T cells with dCas9-SALL1-SDS3 mRNA and synthetic guide RNAs targeting *CXCR3*. Protein depletion was assessed by flow cytometry analysis 24 and 72 h post-nucleofection (Fig. 5D) and compared to transcriptional repression (Fig. 5E). Notably, CXCR3 protein and transcript expression was reduced to ≤10% of basal expression within 24 h of nucleofection and remained robustly repressed 72 h post-nucleofection. These results indicate that dCas9-SALL1-SDS3 mRNA can be co-delivered with synthetic guide RNAs to effect potent, transient repression in therapeutically relevant cells including primary T cells.

### dCas9-SALL1-SDS3 enables functional gene characterization

We have previously demonstrated that synthetic guide RNAs designed for CRISPRa are amenable to arrayed gain-of-function screens in a similar fashion to siRNAs for LOF screening.^52^ To explore whether this novel CRISPRi effector could be used orthogonally to siRNAs for functional gene characterization in arrayed assays, we targeted genes involved in the DNA damage response (DDR) network by siRNA and dCas9-SALL1-SDS3 in parallel. We quantified the impact of target knockdown on the phosphorylation of histone H2AX, an early marker of DNA damage, with high-content analysis, a readout not amenable to a pooled screening, thus highlighting the utility of an arrayed screening approach.

**FIG. 6.**
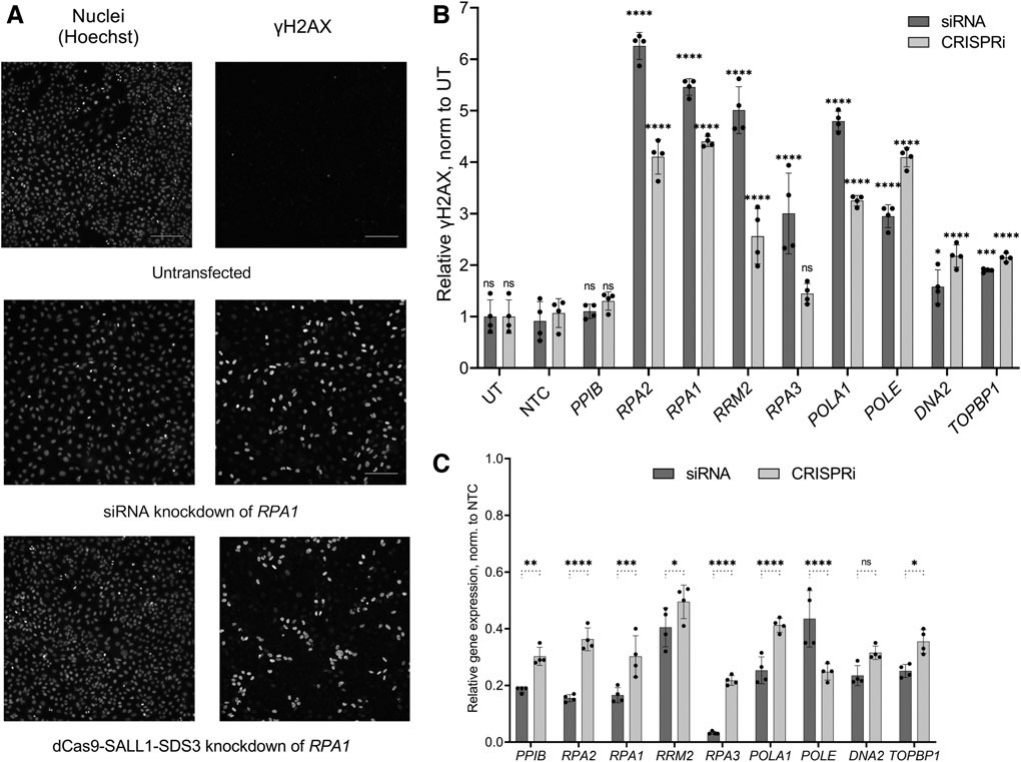
Synthetic sgRNAs can be used in parallel with siRNAs for functional gene characterization. **(A)** Representative immunofluorescent imaging of nuclei (Hoechst, top row) and phosphorylated γH2AX (bottom row) in cells with no manipulation (untreated, left column) or cells following siRNA-mediated knockdown (middle column) or CRISPRi-mediated knockdown (right column) of *RPA1*. **(B)** High-content imaging quantification of phosphorylated γH2AX levels in A549 cells stably expressing dCas9-SALL1-SDS3 72-h post-siRNA- or synthetic CRISPRi sgRNA-targeting of DDR pathway genes. NTC and targeting of a gene not involved in the DDR pathway (PPIB) served as negative controls. All data normalized to UT cells, all statistical comparisons made to the respective NTC. **(C)** Relative mRNA expression of *PPIB* and DDR genes *RPA2, RPA1, RRM2, RPA3, POLA1, POLE, DNA2,* and *TOPBP1* 72 h post-synthetic sgRNA or siRNA delivery in A549 cells stably expressing dCas9-SALL1-SDS3. All data were normalized to the corresponding NTCs. *n* = 4 biologically independent replicates per group. All data presented as mean ± SD. *, **, ***, and ****p < 0.05, 0.01, 0.001, and 0.0001, respectively by two-way ANOVA followed by Šídák's *post hoc* test for multiple comparisons. DDR, DNA damage response.

We selected the p53 WT A549 cell line as a model since the tumor suppressor p53 is a central regulator of the DDR and generated a population of cells stably expressing dCas9-SALL1-SDS3.^68^ We used parallel arrays of synthetic sgRNAs and siRNAs to target eight genes whose knockdown has been shown to impact the cell's DDR.^68^ Cells were stained for phospho-H2AX (Fig. 6A) and subjected to high-content analysis (Fig. 6B) 72 h post-transfection. Upon siRNA knockdown of all eight genes, we found significant increases in the percentages of phospho-H2AX positive cells that ranged from 1.5-fold to 6.2-fold greater than that observed in the UT cells. CRISPRi repression of seven out of the eight targets produced significant increases in phospho-H2AX positive cells ranging from 2.1-fold to 4.4-fold greater than in the UT cells. With both siRNA and CRISPRi, repression of *RPA2* and *RPA1* produced the greatest increases in phospho-H2AX positive cells (greater than fourfold).

For each target, gene knockdown was confirmed by RT-qPCR (Fig. 6C) with siRNA producing between 57% and 97% target mRNA knockdown and CRISPRi leading to 50–78% target mRNA repression. Parallel siRNA and CRISPRi targeting increased the power of our functional characterization, especially for genes such as *DNA2* and *TOPBP1*, whose knockdown produced a less pronounced, though still significant, DDR phenotype (1.5-fold and .2.1-fold increases in phospho-H2AX cells with siRNA and CRISPRi reagents targeting *DNA2*; 1.9-fold and 2.1-fold with siRNA and CRISPRi reagents targeting *TOPBP1*). These results demonstrate that dCas9-SALL1-SDS3 can be used in conjunction with synthetic sgRNAs for arrayed experiments with complex readouts not amenable to pooled screening.

## Discussion

We have discovered and characterized a novel effector for CRISPRi, dCas9-SALL1-SDS3, that can be used to induce transcriptional repression with synthetic sgRNAs at levels more robust than those seen with dCas9-KRAB or dCas9-KRAB-MeCP2. We have shown this approach to be broadly applicable across gene targets and cell types. Indeed, the use of dCas9-SALL1-SDS3 mRNA co-delivered with synthetic sgRNAs results in potent, transient repression in therapeutically relevant models such as hiPSCs and primary T-cells. In addition, the specificity of dCas9-SALL1-SDS3 is like that observed with dCas9-KRAB, making it a highly precise tool for LOF studies.

dCas9-SALL1-SDS3 mediated greater repression than dCas9-KRAB in U2OS cells transfected with synthetic sgRNAs but not sgRNA-expressing plasmids (Fig. 2E). Unlike past work which has focused on the development of improved CRISPRi systems for use with expressed guides, our strategy of screening CRISPRi effectors with synthetic sgRNAs targeting multiple endogenous genes allowed us to identify an effector that outperformed previous generation CRISPRi effectors with this format of guide RNAs.^40,43^ Though dCas9-KRAB-MeCP2 has been reported to exhibit greater repressive activity than dCas9-KRAB when used with expressed guide RNAs, we did not observe consistent improvements in target gene repression when used with synthetic sgRNAs (Fig. 1D–F), possibly because the effector was identified using expressed sgRNAs.

Over the course of our development and characterization of dCas9-SALL1-SDS3, multiple groups reported that the ZIM3 KRAB domain more potently effected target gene repression than the KOX1 KRAB domain used in our study.^41,42^ However, the authors of these studies specifically utilized expressed sgRNAs to identify a more potent KRAB domain. It will be interesting to compare the efficiency of target repression via ZIM3 dCas9-KRAB or dCas9-SALL1-SDS3 using synthetic sgRNAs in future studies.

We have shown via CoIP/MS experiments that dCas9-SALL1-SDS3 recruits Sin3 HDAC complex proteins including SIN3A, HDAC1, SAP30, RBBP4 and RBBP7, all reported to be core complex members.^69,70^ The Sin3 complex mediates transcriptional repression through the deacetylation of TSS-proximal histones.^71–74^ Histone acetylation is a particularly dynamic histone post-translational modification with H3K9, K3K18, and H3K27 hyperacetylation correlated with high transcriptional activity.^75–77^

Surprisingly, we did not observe HDAC1/2 co-precipitation with dCas9-KRAB. The KRAB domain is reported to interact with HDACs indirectly through KAP1 recruitment which could explain the differences between dCas9-SALL1-SDS3 and dCas9-KRAB in our co-precipitation experiments.^78–80^ These data suggest that dCas9-SALL1-SDS3 and dCas9-KRAB are mediating transcriptional repression through the recruitment of distinct repressors complexes and epigenetic modifiers; further work to establish direct interaction partners of dCas9-KRAB and characterization of epigenetic modifications between these systems could help elucidate these differences. As novel CRISPR systems for epigenetic modulation continue to be developed, including systems for long-term heritable epigenetic silencing, it would be of interest to investigate if synthetic sgRNAs could be transiently delivered with these technologies to reprogram the epigenome.^81^

Finally, we have demonstrated that dCas9-SALL1-SDS3 can be used in combination with synthetic sgRNAs in an arrayed format using assays including image-based readouts that are not compatible with screens performed in a pooled format. Our confirmation of a DDR phenotype after knockdown of DDR pathway genes using either siRNA or CRISPRi highlights the utility of the dCas9-SALL1-SDS3 CRISPRi system as an effective orthogonal LOF method. Both techniques confirm the importance of these targeted genes in the cellular response to routine, intrinsic genomic instability and cellular stress (without the introduction of a DNA damaging agent).

Confirmation via two LOF technologies can increase the confidence in functional characterization of unknown targets where knockdown may produce more subtle phenotypic responses. However, more work is needed to explore the subtleties of how transcriptional and post-transcriptional knockdown mechanisms behave in different biological assays. Pooling of multiple synthetic sgRNAs against a gene target abates differences in individual sgRNA activity, increasing the likelihood of a robust phenotypic response, and does not compromise the specificity of dCas9-SALL1-SDS3 targeting. Using this effector in complex, arrayed assays enables mid- to high-throughput screens and the examination of a wide range of phenotypes, comparable to what is currently performed with synthetic siRNA libraries. This combination of complementary LOF tools enhances the ability to interrogate complex biological systems.

## Supplementary Material

Supplemental data

Supplemental data

Supplemental data

Supplemental data

Supplemental data

Supplemental data

Supplemental data

Supplemental data

Supplemental data

Supplemental data

Supplemental data

Supplemental data

## Exclusivity Statement

Portions of Figure 2, Supplementary Figure 2, and Supplementary Figure 7 have been shared in similar form at the following conferences: Keystone Precision Engineering of the Genome, Epigenome and Transcriptome, virtual, Mar 2021; CRISPR2021, virtual, 2021; ISSCR, virtual, Jun 2021; CSH Genome Engineering: CRISPR Frontiers, virtual, Aug 2021; CRISPR2.0, virtual, Nov 2021; ASCR, virtual, 2021; Keystone Organoids, Keystone, Co, Apr 2022; Keystone Precision Genome Engineering, Keystone, Co, 2022; RNA society, Boulder, May 31-Jun 5, 2022; CRISPR2022, Boston, Jun 2022; ISCCR; San Francisco, Jun 2022; Keystone Gene Regulation, Santa Fe, NM, Jun 2022.
