# Supplementary material for: A Novel CRISPR Interference Effector Enabling Functional Gene Characterization with Synthetic Guide RNAs
Source: CRISPR J. 2022 Dec 12;5(6):769–86. doi: 10.1089/crispr.2022.0056 (PMC9805873; doi:10.1089/crispr.2022.0056)
Supplement: Supplemental data [file Supp_FigS1.pdf]

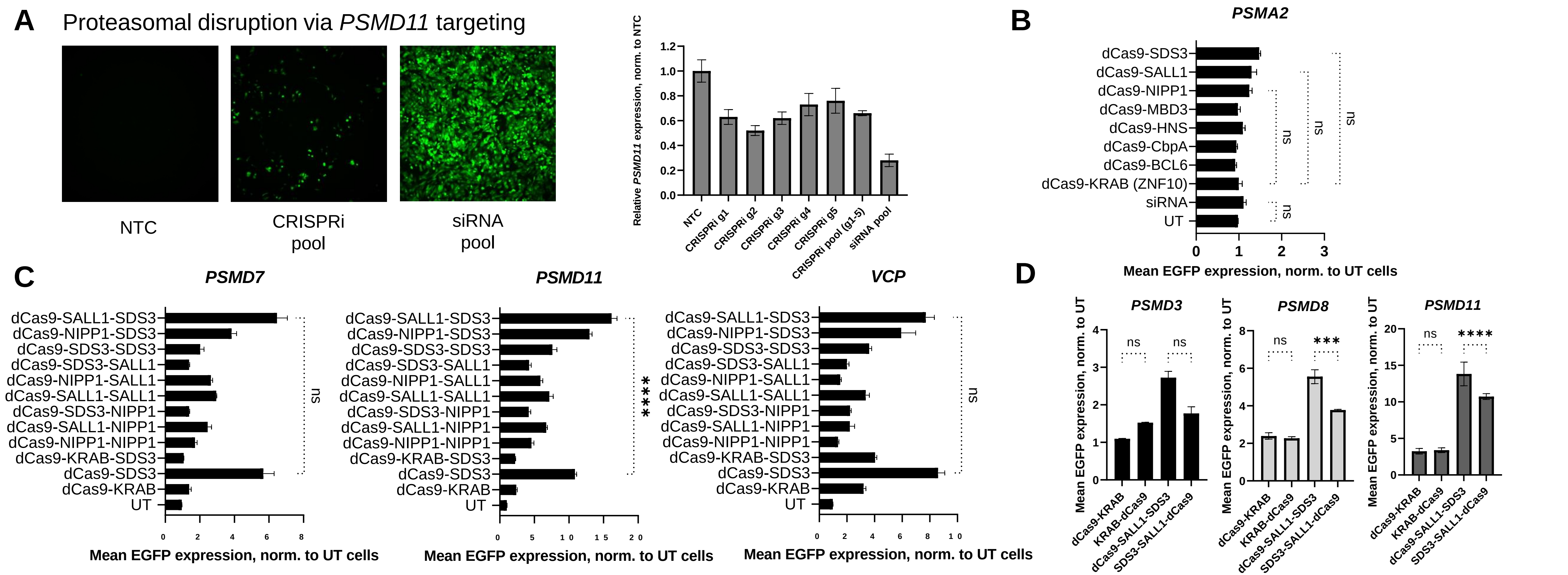

Supplemental Figure 1: Identification of a potent effector for CRISPRi applications

A) Representative imaging (left) of U2OS Ubi[G76V]-EGFP cells (proteasome assay) stably expressing dCas9-KRAB induced by targeting *PSMD11* with either a pool of 4 siRNAs or a pool of 4 synthetic crRNA:tracrRNAs designed for CRISPRi as compared to cells transfected with non-targeting controls (NTC). Relative mRNA expression (right) of *PSMD11* 72 hours post-transfection with either individual or pooled synthetic guide RNAs designed for CRISPRi, or a pool of siRNAs. All data were normalized to the corresponding NTC.

B) Mean EGFP fluorescence induced by targeting *PSMA2* with synthetic sgRNAs in U2OS Ubi[G76V]-EGFP cells stably expressing dCas9 fused to single repressor domains. siRNA delivery was used as a positive control for fluorescence induction. Mean fluorescence was measured 72 hours post-transfection and is shown relative to untransfected (UT) cells.

C) Mean EGFP fluorescence induced by targeting candidate proteasome-related genes *PSMD7*, *PSMD11*, and *VCP* with synthetic sgRNAs in U2OS Ubi[G76V]-EGFP cells stably expressing dCas9 fused to bipartite repressor domains. Mean fluorescence was measured 72 hours post-transfection and is shown relative to untransfected (UT) cells.

D) Mean EGFP fluorescence induced by targeting proteasomal genes *PSMD3*, *PSMD8*, and *PSMD11*with synthetic sgRNAs in Ubi[G76V]-EGFP cells stably expressing dCas9-KRAB, KRAB-dCas9, dCas9-SALL1-SDS3, or SDS3-SALL1-dCas9. Mean fluorescence was measured 72 hours post-transfection and is shown relative to untransfected (UT) cells.

N = 3 biological independent replicates per group. All data presented as mean ±S.D. *** and **** $p < 0.001$ and 0.0001, respectively by one or two-way ANOVA followed by Tukey's post hoc test for multiple comparisons.
