# Supplementary material for: A Novel CRISPR Interference Effector Enabling Functional Gene Characterization with Synthetic Guide RNAs
Source: CRISPR J. 2022 Dec 12;5(6):769–86. doi: 10.1089/crispr.2022.0056 (PMC9805873; doi:10.1089/crispr.2022.0056)
Supplement: Supplemental data [file Supp_FigS2.pdf]

A

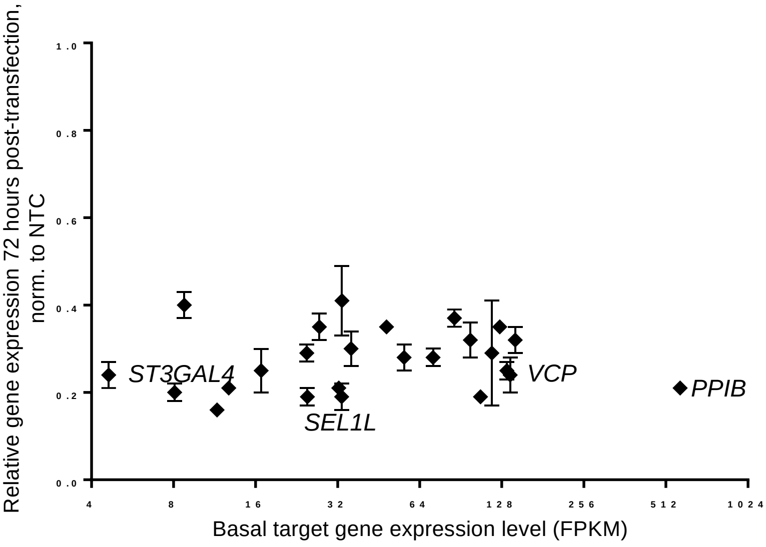

B

Gene Target	Basal gene expression (FPKM)	Relative gene expression 72 hours post-transfection
SOX2	4.62312	0.24
ST3GAL4	8.07584	0.2
POLA1	8.76342	0.4
BRCA1	11.5563	0.16
MRE11A	12.7585	0.21
SETD3	16.7743	0.25
RPA1	24.6688	0.29
HBP1	24.7715	0.19
RPA2	27.4282	0.35
CD46	32.2929	0.21
SEL1L	33.1022	0.19
KIF11	33.1645	0.41
PSMD11	35.8155	0.3
LBR	48.3418	0.35
PSMD7	56.0718	0.28
TFRC	71.5898	0.28
PSMA2	85.7713	0.37
PSMD3	98.1256	0.32
PSMD8	106.902	0.19
CBX1	117.658	0.29
CANX	125.784	0.35
VCP	133.626	0.25
CDC20	137.472	0.24
CD151	143.435	0.32
PPIB	577.35	0.21

C

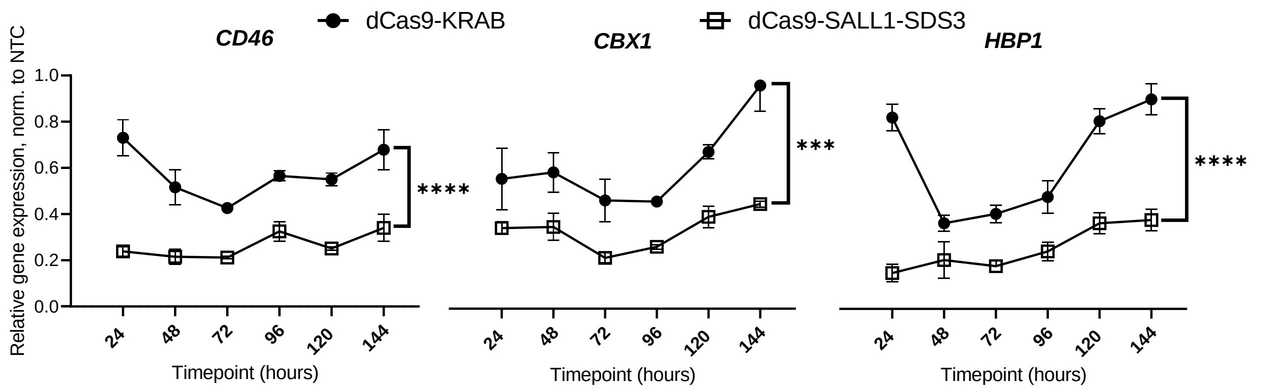

D

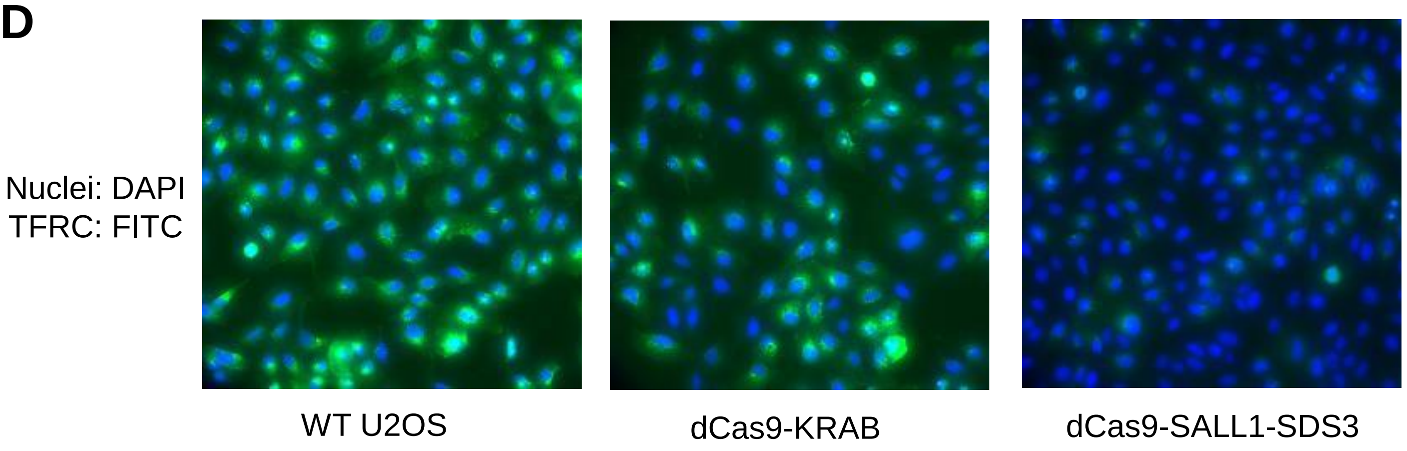

E

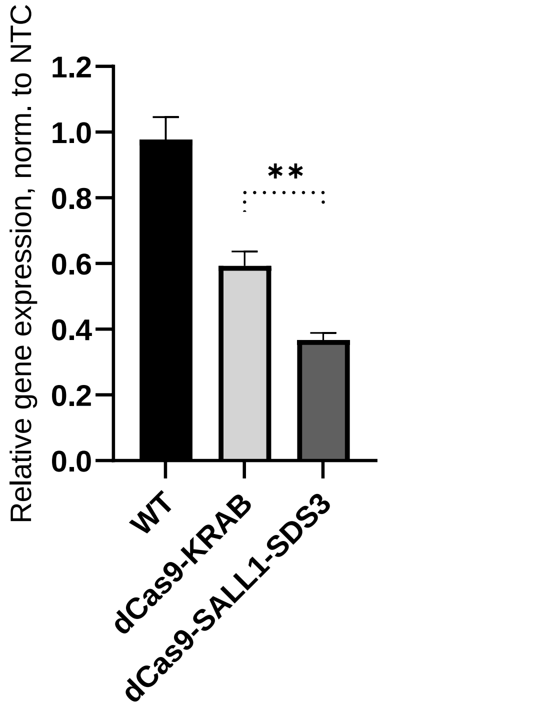

F

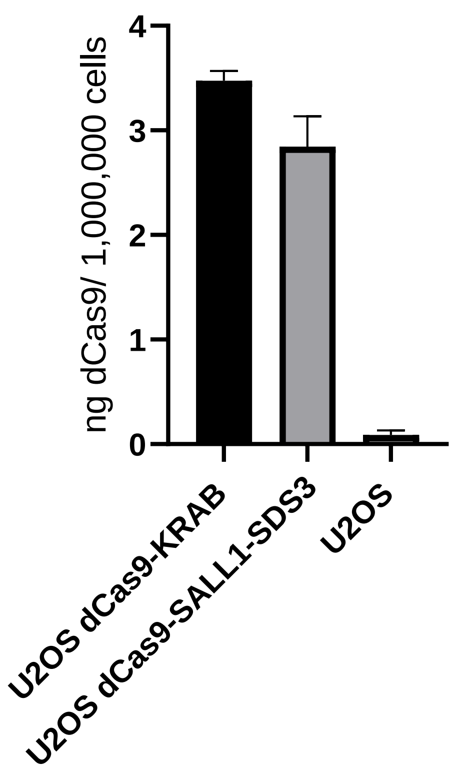

Supplemental Figure 2: Characterization of dCas9-SALL1-SDS3-mediated repression

a) Correlation of dCas9-SALL1-SDS3-mediated transcriptional repression 72 hours post-transfection of synthetic sgRNAs and basal transcript expression levels of each targeted gene in U2OS cells, shown as fragments per kilobase million (FPKM). FPKM was obtained from a whole transcriptome RNA-seq dataset. All data were normalized to the corresponding non-targeting controls.
b) Table showing basal gene expression and relative gene expression 72 hours post-transfection of pooled synthetic sgRNAs for each gene targeted in panel A.
c) Comparison of relative mRNA expression of *CD46*, *CBX1,*and *HBP1* in U2OS cells stably expressing either dCas9-SALL1-SDS3 or dCas9-KRAB 24, 48, 72, 96, 120, and 144 hours post-transfection of synthetic sgRNAs targeting the respective genes. All data were normalized to the corresponding NTCs. Repeated measures ANOVA was performed with *** and **** $p < 0.001$ and 0.0001, respectively.
d) Representative TFRC immunostaining of wild-type (WT) U2OS cells (left) or U2OS cells stably expressing either dCas9-KRAB (center) or dCas9-SALL1-SDS3 (right) 96 hours post-transfection of pooled synthetic sgRNAs targeting *TFRC*. Cells were fixed, permeabilized, and blocked 96 hours post-transfection. Cells were stained with a primary antibody targeting TFRC and an Alexa Fluor 488 conjugated secondary antibody; Hoechst was used to identify nuclei.
e) Relative *TFRC* mRNA expression 96 hours post-transfection of pooled sgRNAs targeting *TFRC* in U2OS cells stably expressing dCas9-KRAB or dCas9-SALL1-SDS3. All data were normalized to the corresponding NTC.
f) Mean dCas9 expression assessed by ELISA in U2OS cells stably expressing dCas9-KRAB or dCas9-SALL1-SDS3 or the WT U2OS cell line. N=2 biologically independent samples.

N = 3 biological independent replicates per group unless otherwise noted. All data presented as mean ±S.D. ** $p < 0.01$ by one-way ANOVA followed by Tukey's post hoc test for multiple comparisons unless otherwise noted.
