# Supplementary material for: A Novel CRISPR Interference Effector Enabling Functional Gene Characterization with Synthetic Guide RNAs
Source: CRISPR J. 2022 Dec 12;5(6):769–86. doi: 10.1089/crispr.2022.0056 (PMC9805873; doi:10.1089/crispr.2022.0056)
Supplement: Supplemental data [file Supp_FigS3.pdf]

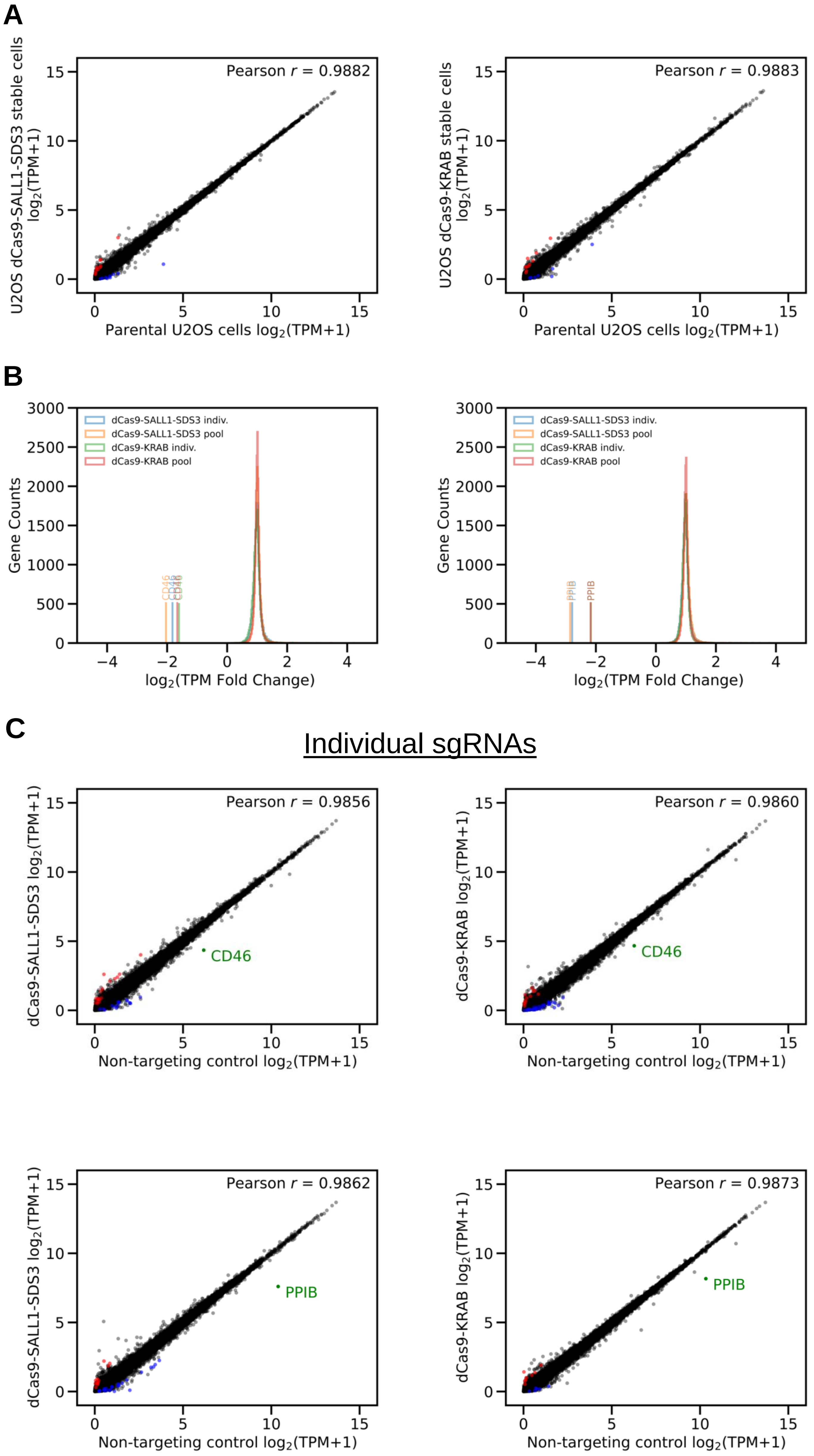
A
Pearson r = 0.9882
U2OS dCas9-SALL1-SDS3 stable cells log2(TPM+1)
Parental U2OS cells log2(TPM+1)
Pearson r = 0.9883
U2OS dCas9-KRAB stable cells log2(TPM+1)
Parental U2OS cells log2(TPM+1)
B
dCas9-SALL1-SDS3 indiv.
dCas9-SALL1-SDS3 pool
dCas9-KRAB indiv.
dCas9-KRAB pool
Gene Counts
log2(TPM Fold Change)
CD46
PPIB
C
Individual sgRNAs
Pearson r = 0.9856
dCas9-SALL1-SDS3 log2(TPM+1)
Non-targeting control log2(TPM+1)
CD46
Pearson r = 0.9860
dCas9-KRAB log2(TPM+1)
Non-targeting control log2(TPM+1)
CD46
Pearson r = 0.9862
dCas9-SALL1-SDS3 log2(TPM+1)
Non-targeting control log2(TPM+1)
PPIB
Pearson r = 0.9873
dCas9-KRAB log2(TPM+1)
Non-targeting control log2(TPM+1)
PPIB

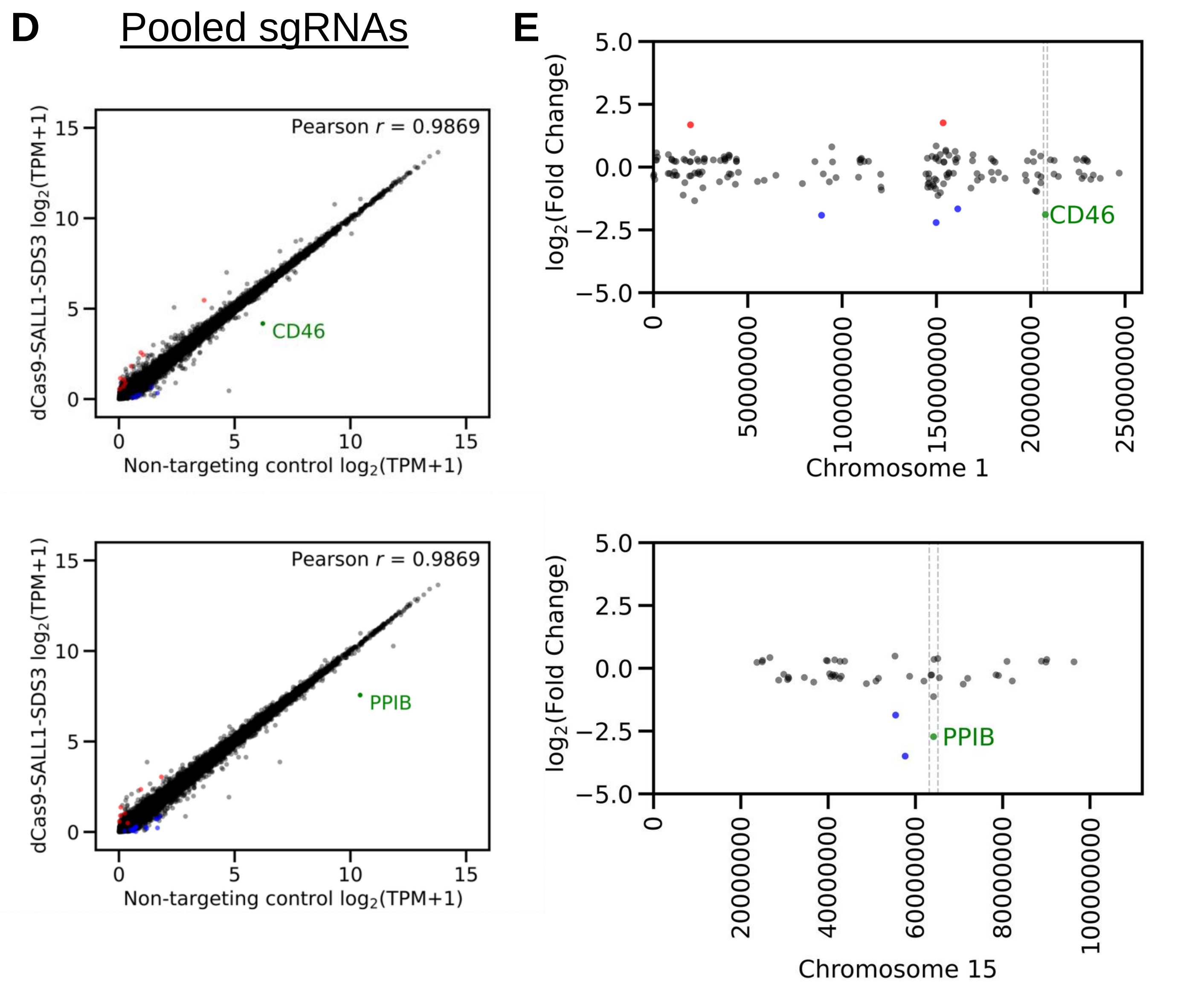

Supplemental Figure 3: dCas9-SALL1-SDS3 mediates highly specific transcriptional repression

A) Scatter plots of gene expression levels (log2 transformed TPM+1, Transcripts Per Million with a pseuudocount of 1 added prior to log transformation) in U2OS cells stably expressing dCas9-SALL1-SDS3 (left) or dCas9-KRAB (right) compared to parental (wild-type) U2OS cells (x-axis). DESeq analysis was used to identify up- and down-regulated genes. Up- or down-regulated genes ($p < 0.05$) with absolute log2 fold change >1.5 in gene expression (represented as log2 transformed TPM+1) marked red and blue, respectively. R indicates Pearson's correlation coefficient, calculated for log-transformed values on all genes.

B) Histogram showing the distribution of global transcriptional expression, represented as Transcripts Per Million (TPM), in U2OS cells stably expressing either dCas9-SALL1-SDS3 or dCas9-KRAB that were transfected with either individual (indiv.) or pooled synthetic sgRNAs targeting *CD46* (left) or *PPIB* (right). Fold change in *CD46* or *PPIB* and global transcriptional expression compared to the corresponding non-targeting control (NTC) is denoted on the x-axis and represented as $\log_2$ transformed TPM fold change. N=2 biologically independent samples per group.

C) Scatter plots of gene expression levels in U2OS cells stably expressing dCas9-SALL1-SDS3 (left) or dCas9-KRAB (right) transfected with individual synthetic sgRNA targeting *CD46* (top) or *PPIB* (bottom) compared to cells transfected with NTCs.

D) Scatter plots of gene expression levels in U2OS cells stably expressing dCas9-SALL1-SDS3 transfected with pooled synthetic sgRNAs targeting *CD46* (top) or *PPIB* (bottom) compared to cells transfected with NTCs.

E) Genomic mapping of significantly differentially expressed genes ($p < 0.05$) across chromosome 1 (top) or chromosome 15 (bottom) in U2OS cells stably expressing dCas9-SALL1-SDS3 that were transfected with individual synthetic sgRNA targeting *CD46* (top) or *PPIB* (bottom). Up- or down-regulated genes with absolute log2 fold change >1.5 in gene expression (represented as log2 transformed TPM+1) marked red and blue, respectively. The 1 Mb region up and downstream of the sgRNA target site is denoted with dotted lines.
