# Supplementary material for: A Novel CRISPR Interference Effector Enabling Functional Gene Characterization with Synthetic Guide RNAs
Source: CRISPR J. 2022 Dec 12;5(6):769–86. doi: 10.1089/crispr.2022.0056 (PMC9805873; doi:10.1089/crispr.2022.0056)
Supplement: Supplemental data [file Supp_FigS4.pdf]

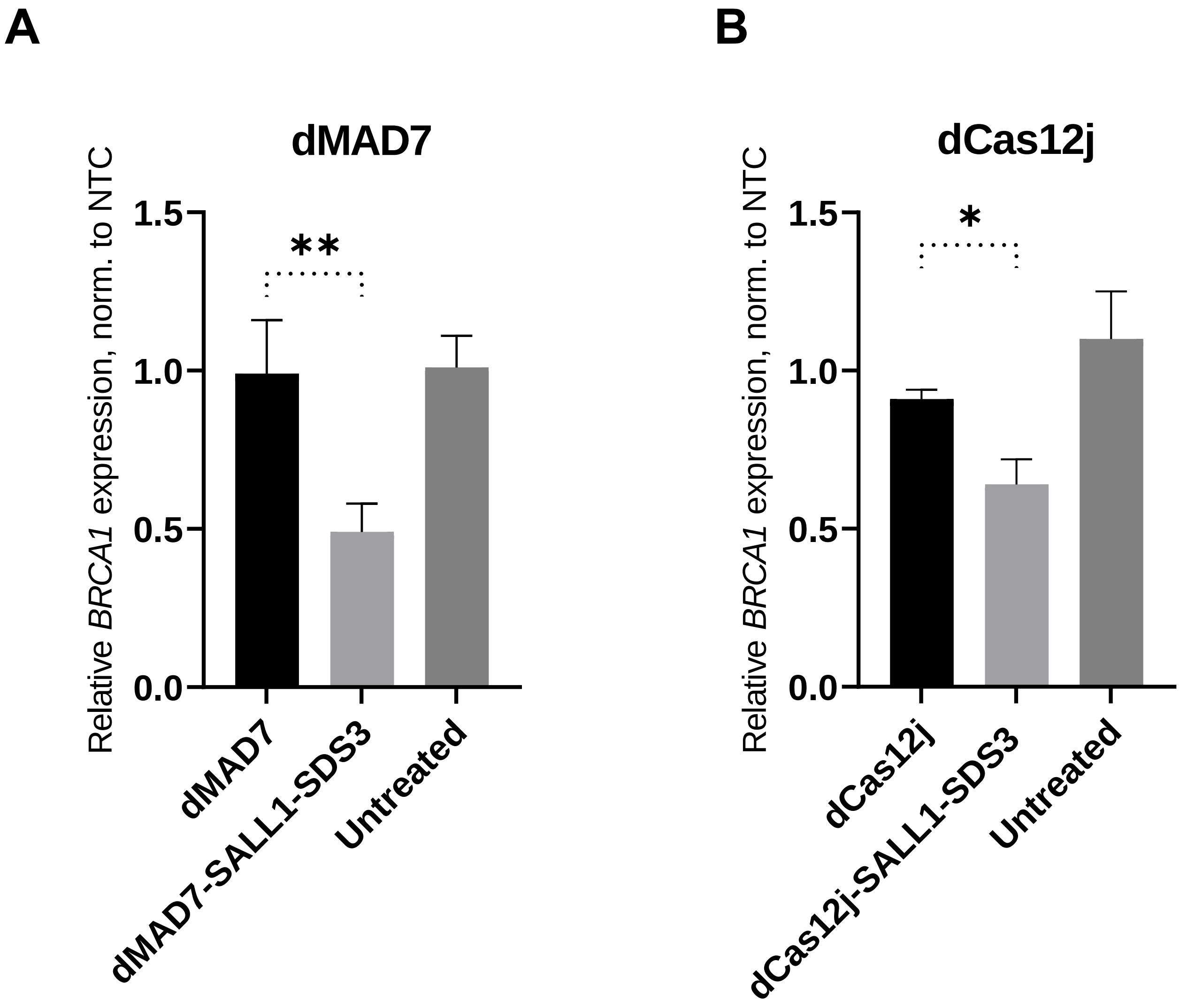

Supplemental Figure 4: SALL1-SDS3-mediated repression with Type V Cas enzymes

A and B) Relative mRNA expression 48-hours post-transfection of individual crRNAs targeting *BRCA1* in U2OS cells stably expressing dMAD7 or dMAD7-SALL1-SDS3 (left panel) or dCas12j or dCas12j-SALL1-SDS3 (right panel). All data were normalized to the corresponding non-targeting control. N = 3 biological independent replicates per group. Data presented as mean ± S.D. *and ** $p < 0.05$ and 0.01, respectively by one-way ANOVA followed by Tukey's post hoc test for multiple comparisons.

dMAD7 crRNA sequence, target region in bold:

UUAAUUUCUACUCUUGUAGAU**UCAGAUAACUGGGCCCCUGCG**

dCas12j crRNA sequence, target region in bold:
AAUAGAUUGCUCCUUACGAGGAGAC**CUGGACGGGGGACA**
