# Supplementary material for: A Novel CRISPR Interference Effector Enabling Functional Gene Characterization with Synthetic Guide RNAs
Source: CRISPR J. 2022 Dec 12;5(6):769–86. doi: 10.1089/crispr.2022.0056 (PMC9805873; doi:10.1089/crispr.2022.0056)
Supplement: Supplemental data [file Supp_FigS5.pdf]

A

Enriched interactions in dCas9-SALL1-SDS3 Co-IPs vs WT

Gene names	Log2 fold change	-log(p value)	p value	Adjusted p value
**dCas9-SALL1-SDS3**	**5.717**	**6.791**	**1.620E-07**	**0.00014**
SIN3A	***4.403***	***5.776***	***1.675E-06***	***0.00075***
RBBP7	***2.521***	***5.092***	***8.086E-06***	***0.00241***
SAP30L	***6.642***	***4.865***	***1.364E-05***	***0.00275***
SAP130	***5.244***	***4.810***	***1.551E-05***	***0.00275***
HDAC1	***2.447***	***4.734***	***1.845E-05***	***0.00275***
ING2	***2.356***	***4.452***	***3.532E-05***	***0.00451***
SAP30	***4.658***	***4.251***	***5.609E-05***	***0.00627***
BRMS1L	***5.433***	***3.991***	***1.022E-04***	***0.01015***
RBBP4	***1.675***	***3.784***	***1.644E-04***	***0.01470***
**LIG3**	**1.471**	**3.524**	**2.992E-04**	**0.02432**
NEGR1	2.808	3.000	1.000E-03	0.06880
TAT	4.260	2.717	1.920E-03	0.10588
PINX1	1.096	2.696	2.013E-03	0.10588

B

Enriched interactions in dCas9-SALL1-SDS3 Co-IPs vs dCas9

Gene names	Log2 fold change	-log(p value)	p value	Adjusted p value
SAP30L	***5.965***	***6.270***	***5.369E-07***	***0.00017***
SAP130	***4.838***	***6.235***	***5.826E-07***	***0.00017***
BRMS1	***4.222***	***5.996***	***1.009E-06***	***0.00023***
HDAC1	***2.848***	***5.400***	***3.980E-06***	***0.00071***
SAP30	***5.131***	***4.433***	***3.691E-05***	***0.00551***
BRMS1L	***5.494***	***4.265***	***5.429E-05***	***0.00571***
RBBP4	***1.530***	***4.241***	***5.736E-05***	***0.00571***
RBBP7	***2.718***	***4.049***	***8.935E-05***	***0.00801***
SIN3A	***5.720***	***3.416***	***3.838E-04***	***0.02972***
HDAC2	***1.816***	***3.400***	***3.980E-04***	***0.02972***
ING2	*2.512*	*3.073*	*8.444E-04*	*0.05404*
IFI16	1.003	2.810	1.550E-03	0.09261
CHMP4B	1.126	2.710	1.951E-03	0.10926
RBM28	0.941	2.261	5.477E-03	0.21337

Supplemental Figure 5: dCas9-SALL1-SDS3 specifically interacts with the SIN3A complex

A and B) Tables of enriched interactions in dCas9-SALL1-SDS3 Co-IPs vs wild-type (WT) (A) or dCas9 (B). Bold text denotes significant interactions. Red italicized text denotes components of the SIN3 complex.
