# Supplementary material for: A Novel CRISPR Interference Effector Enabling Functional Gene Characterization with Synthetic Guide RNAs
Source: CRISPR J. 2022 Dec 12;5(6):769–86. doi: 10.1089/crispr.2022.0056 (PMC9805873; doi:10.1089/crispr.2022.0056)
Supplement: Supplemental data [file Supp_FigS6.pdf]

A

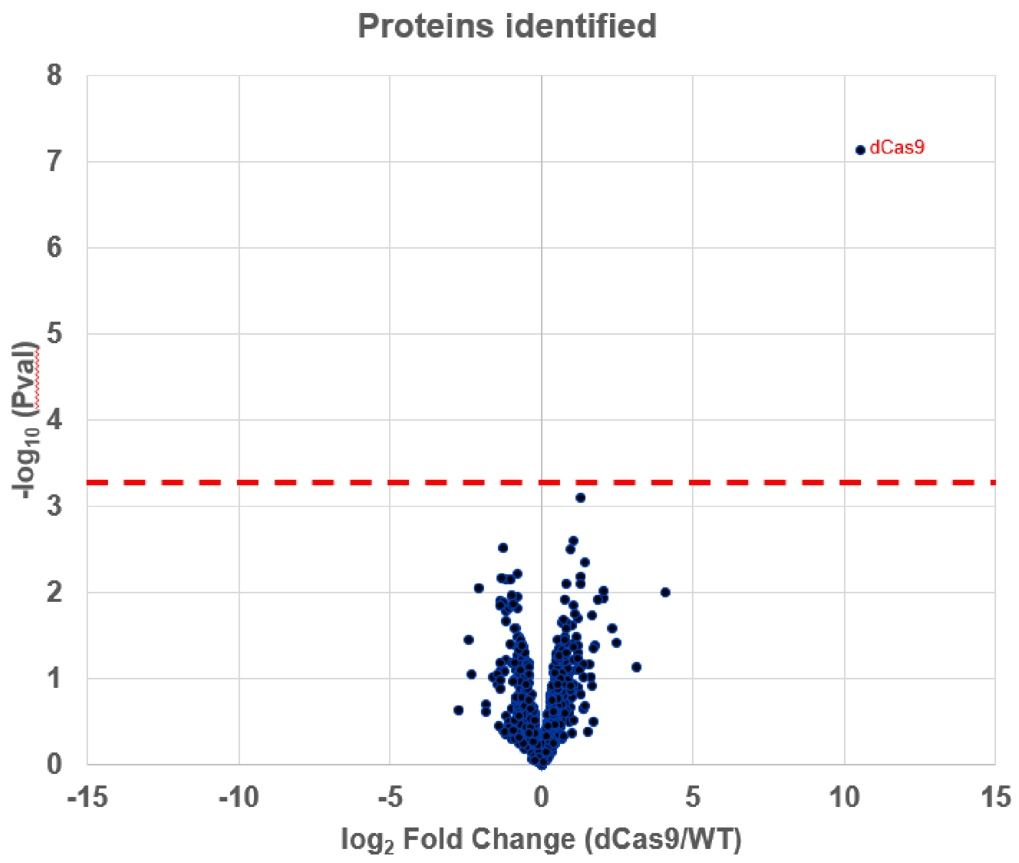

B

Enriched interactions in dCas9 Co-IPs vs WT

Gene names	Log2 fold change	-log(p value)	p value	Adjusted p value
**dCas9**	**10.539**	**7.135**	**7.333E-08**	**0.00007**
CHERP	1.298	3.094	8.060E-04	0.36147
DSP	4.102	2.000	1.000E-02	0.42751
UBA52	2.066	2.017	9.618E-03	0.42751
CWC15	2.051	1.941	1.144E-02	0.42751
CALD1	1.843	1.911	1.226E-02	0.42751
KIF11	1.417	2.346	4.508E-03	0.42751
SSB	1.305	2.189	6.465E-03	0.42751
FAM32A	1.278	2.102	7.913E-03	0.42751
VDAC2	1.074	1.848	1.419E-02	0.42751
MRPS27	1.055	2.605	2.485E-03	0.42751
LIG3	0.957	2.507	3.109E-03	0.42751

C

Enriched interactions in WT Co-IPs vs dCas9

Gene names	Log2 fold change	-log(p value)	p value	Adjusted p value
NOM1	-2.062	2.057	8.770E-03	0.42751
URB2	-1.383	1.845	1.430E-02	0.42751
HNRNPA1;HNRNPA1L2	-1.350	1.903	1.249E-02	0.42751
ZNF787	-1.336	1.846	1.425E-02	0.42751
BRD1	-1.318	2.163	6.865E-03	0.42751
PWP1	-1.299	1.845	1.429E-02	0.42751
PRPF4B	-1.282	2.513	3.072E-03	0.42751
CHMP4B	-1.272	1.880	1.317E-02	0.42751
STK38	-1.158	2.150	7.087E-03	0.42751
MRTO4	-1.024	2.151	7.057E-03	0.42751
RRP9	-0.997	1.943	1.141E-02	0.42751

Supplemental Figure 6: dCas9 does not significantly interact with the mammalian proteome

A) Volcano plot of enriched protein interactions in dCas9 vs wild-type (WT) Co-IPs, identified by mass spectrometry. Significant interactions were defined at a threshold of $p<0.0008$ (adjusted p-value <0.05), denoted by horizontal dashed line, respectively. N=4 (dCas9) and N=3 (WT) biological replicates per group.

B and C) Tables of enriched interactions in dCas9 Co-IPs vs WT (B), or WT Co-IPs vs dCas9 (C). Bold text denotes significant interactions.
