# Supplementary material for: A Novel CRISPR Interference Effector Enabling Functional Gene Characterization with Synthetic Guide RNAs
Source: CRISPR J. 2022 Dec 12;5(6):769–86. doi: 10.1089/crispr.2022.0056 (PMC9805873; doi:10.1089/crispr.2022.0056)
Supplement: Supplemental data [file Supp_FigS7.pdf]

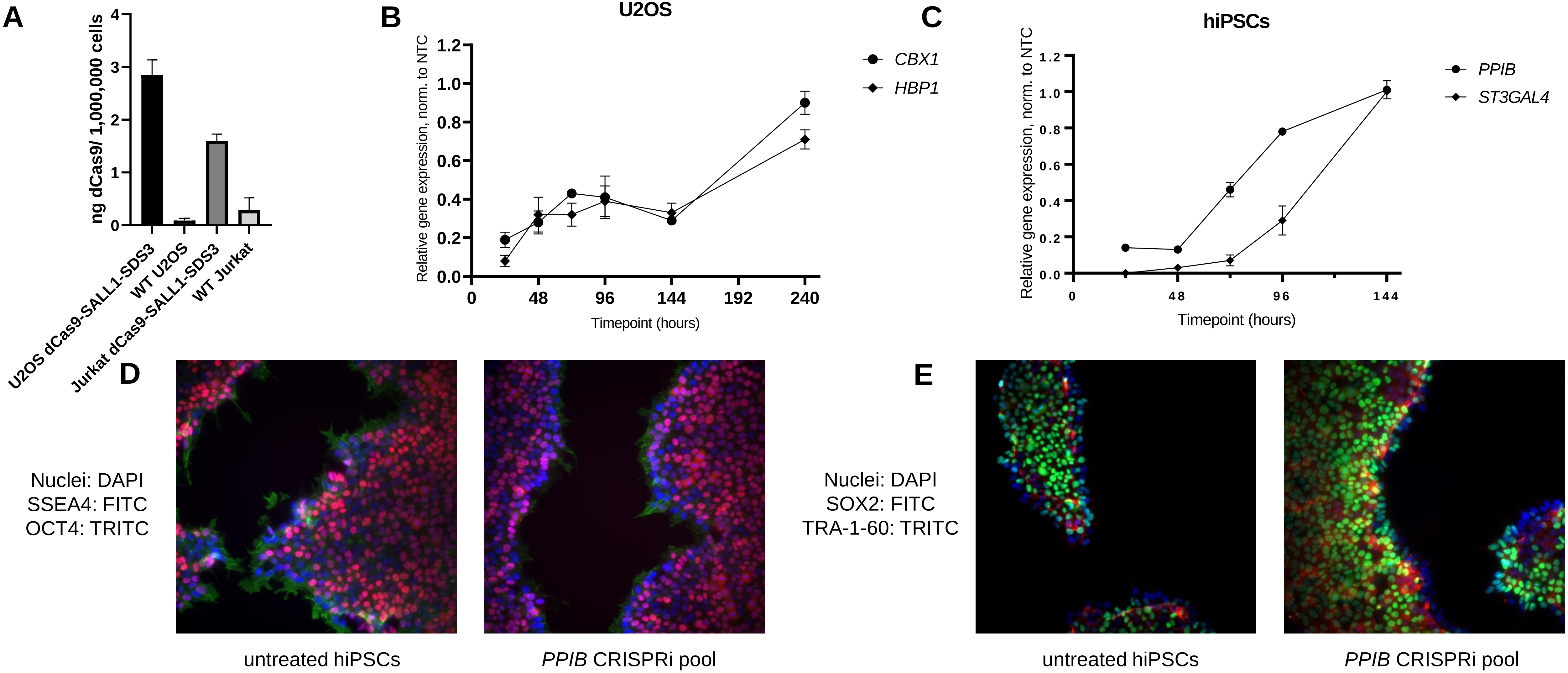

Supplemental Figure 7: Potent, transient repression with in-vitro-transcribed dCas9-SALL1-SDS3 mRNA and synthetic sgRNAs

A) Mean dCas9 expression assessed by ELISA in U2OS and Jurkat cells stably expressing dCas9-SALL1-SDS3 alongside expression in wild-type U2OS and Jurkat lines. N= 2 biologically independent samples. B and C) Relative mRNA levels of denoted target genes over time in U2OS cells (B) or human induced pluripotent stem cells (hiPSCs) (C) following co-delivery of dCas9-SALL1-SDS3 mRNA and synthetic sgRNAs. All data were normalized to the corresponding non-targeting controls (NTC). N = 3 biologically independent replicates.

D and E) Representative SSEA4/OCT4 (left) and SOX2/TRA-1-60 (right) immunostaining of untreated hiPSCs or hiPSCs 168 hours post-nucleofection with dCas9-SALL1-SDS3 mRNA and pooled synthetic sgRNAs targeting *PPIB*. Cells were fixed, permeabilized, and blocked 168 hours post-nucleofection. Cells were co-stained for pluripotency markers SSEA and OCT4 (left) and SOX2 and TRA-1-60 (right) using Invitrogen™ Pluripotent Stem Cell 4-Marker Immunocytochemistry Kit (Cat A24881, ThermoFisher) following manufacturer's protocol. Hoechst was used to identify nuclei.
